# Chemical composition, antioxidant, anti-inflammatory, and cytotoxic activities of *Opuntia stricta* cladodes

**DOI:** 10.1371/journal.pone.0209682

**Published:** 2019-01-29

**Authors:** Ogochukwu Izuegbuna, Gloria Otunola, Graeme Bradley

**Affiliations:** 1 Department of Biochemistry, Faculty of Science & Agriculture, University of Fort Hare, Alice, South Africa; 2 Medicinal Plants and Economic Development (MPED) Research Centre, Department of Botany, Faculty of Science & Agriculture, University of Fort Hare, Alice, South Africa; University of Palermo, ITALY

## Abstract

**Background:**

The *Opuntia spp*. have been used in traditional medicine for many centuries. It is used in the management of diseases that involves oxidative stress, especially diabetes, obesity and cancer. *Opuntia stricta* (Haw) is one of the relatively unknown species in South Africa where it is regarded more as a weed. Because of this, not much is known about its chemical composition.

**Aim:**

To determine the chemical composition, antioxidant, anti-inflammatory, and cytotoxic activities of *Opuntia stricta* cladodes.

**Methods:**

The phytochemical composition of acetone, aqueous and ethanol extract of cladodes of *Opuntia stricta* (Haw), as well as the vitamins A, C and E of its dried weight cladodes and the antioxidant activities, were evaluated using standard *in vitro* methods. The anti-inflammatory and cytotoxic activities were evaluated using cell-based assays. The phytochemical composition and vitamins were determined spectrophotometrically, while the antioxidant activities were determined by DPPH, nitric oxide, hydrogen peroxide scavenging activity and phosphomolybdenum (total) antioxidant activity. Anti-inflammatory activity was determined using RAW 264.7 cells, while cytotoxicity was determined using U937 cells.

**Results:**

The phytochemical composition showed a significant difference in the various extracts. The total phenolics were higher than other phytochemicals in all the extracts used. All the extracts displayed antioxidant activity, while most of the extracts showed anti-inflammatory activity. Only one extract showed cytotoxicity, and it was mild.

**Conclusion:**

The results show that the *Opuntia stricta* is rich in polyphenolic compounds and has good antioxidant activity as well as anti-inflammatory activities.

## Introduction

Oxidative stress (OS) occurs as a result of an imbalance between generated reactive metabolites also known as reactive oxygen species (ROS) and the body's antioxidant system. It is a normal physiological condition created to maintain redox homeostasis. However, persistence in the imbalance can cause cellular damage and eventually disease. ROS are known to act on some signalling pathways, modulating physiological responses [1]. ROS are generated through the electron transport chain in the mitochondria, and the cytochrome P450 [2]. Proteins and lipids are some of the major targets for attack, and their modification can lead to some diseases [3]. ROS has been linked to a number of diseases, most of which are chronic diseases. They include atherosclerosis[4], cardiovascular diseases[5], diabetes[6], inflammatory diseases [7], cancer[8] etc. Most of these diseases have a background inflammation, which is chronic in nature and involves the release of ROS. In some cancers, ROS is known to promote cell survival and proliferation [9] as well as play a role in drug resistance [10]. ROS is equally involved in the expression of inflammatory markers [11] some of which play a role in cell proliferation and metastasis[12] as well as mediate immunity [13].

Since ROS are very important in cellular homeostasis and body physiology, regulation of ROS via the body's endogenous antioxidants is a safe means to keep ROS production in check. However when this fails (which happens in diseases), reversing the process through other means becomes very important. Over the years antioxidant supplementation has been used for the prevention and management of ailments caused by oxidative stress[14]. Some of these natural compounds that are polyphenols and vitamins affect many biological processes. They interact with ROS and other inflammatory mediators, modulating their activities to prevent cellular stress [15] which can lead to cellular transformation and eventual cancer [16]. These natural compounds are found in plants and include Vitamins C,E and D, the carotenoids and complex polyphenols. Their mechanism of actions includes inhibition of the catalytic enzymes involved in ROS production, Scavenging of ROS, and upregulation of endogenous antioxidant defence [17]. Vitamins have also been shown to play a role in inhibiting ROS production especially in cancers [18]. For example, vitamin C was reported to induce differentiation and death of acute myeloid leukaemia cells in both *in vitro* and orthotopically transplanted mice[19][20].

The need for powerful antioxidants and anti-inflammatory agents to inhibit the process of cellular transformation have made inroads into plants and herb sources. Phenolic compounds and flavonoids are reported as having excellent antioxidant properties [21].

*Opuntia spp*. represent one of the most diverse and distributed genera of plants. It is found on all continents except Antarctica[22]. It has its highest degree of diversity in Mexico, where it probably originated, with various degree of domestication observed. It has since been introduced all over the world and can be found in temperate, subtropical and tropical regions. It was introduced into South Africa in the 1700s where it is regarded as a weed and has been actively controlled using biological means. It is a member of the family Cactaceae, subfamily Opuntioideae, tribe Opuntieae. About 1500 species of cactus are in the genus Opuntia. The plants (especially *Opuntia ficus indica*) are known as health-promoting foods with their sweet, edible fruits and young cladodes eaten as a vegetable in salads; and also some medicinal properties[23].

*Opuntia spp*. extracts have been used for several centuries in the management of different ailments which include chronic and inflammatory conditions like diabetes, rheumatism, asthma, hypercholesterolaemia, and hypertension[24]. Recent scientific studies have further increased interests in these plants. Mice fed with methanolic extract of *Opuntia joconostle* seeds showed a significantly lowered plasma LDL cholesterol and triglycerides levels compared to animals fed with placebo [25]. Although it has not been reported to be used as an antineoplastic agent in traditional medicine, scientific studies carried out showed Opuntia has some activity on cancer. Work done by Kim and colleagues revealed that extracts from *Opuntia humifusa* cladodes could cause apoptosis in MCF-7 cells and human colonSW-480 cells[26]. Water partitioned fractions of stem and fruits of *Opuntia humifusa* has also been reported to inhibit the growth of U87MG glioblastoma cells with increased production of reactive oxygen species in the cells [27].

While several species of the family *Opuntiaceae* have been investigated, there is a dearth of information on the biochemical properties of *Opuntia stricta* (phytochemical composition, essential oils, antioxidant, anti-inflammatory, cytotoxic activities). This study aimed to estimate the total phenol, flavonoid, flavonol, proanthocyanidin, tannins, alkaloids, saponins and phytate contents in the water, acetone and ethanol extracts of *Opuntia stricta* cladodes as well as the vitamins A,E and C content and essential oils composition. The study also investigated the antioxidant, anti-inflammatory and the cytotoxic profile of the plant in order to justify its traditional use and add to the body of knowledge.

*Opuntia stricta* cladodes in this study were discovered to have all the phytochemicals investigated for. It equally showed good antioxidant activities when compared with the standards in the various experiments. It showed comparable anti-inflammatory activity to Celecoxib, a selective COX-2 inhibitor. However its cytotoxic activity was mild against the cell lines used. The essential oils showed it has compounds with antioxidant, anti-inflammatory and cytotoxic activities.

## Materials and methods

The cladodes of *Opuntia stricta* were collected from the plant growing within the University of Fort Hare campus in Alice, South Africa. The plant was authenticated by Tony Dolds at the Albany herbarium in Rhodes University, Grahamstown, South Africa. The cladodes were oven-dried at 40°C and pulverized using a milling machine. About 300g of each of the pulverized samples was extracted separately with 1L of each of the solvents, water, ethanol and acetone for 48 h. The extracts were filtered through Whatman No. 1 filter paper and evaporated to dryness under reduced pressure at different temperatures using a rotary evaporator. The filtrate of aqueous extract obtained was quickly frozen at -40°C and dried for 48 h using a freeze dryer (Savant Refrigerated vapour Trap, RV T41404, USA). The extracts were stored away in a refrigerator at 4°C.

### Phytochemical analyses

#### Estimation of total phenol content

The total phenol was estimated spectrophotometrically by using the Folin-Ciocalteu assay method[28]. Here 0.5ml of the extract was added to 2.5ml of 10% Folin-Ciocalteu reagent in tubes. It was then vortexed for 30s and allowed to stand for 10 min at 25°C. 2 ml of 7.5% anhydrous sodium carbonate was added to the solution and vortexed again for another 30s. The tubes were incubated in a water bath at 40°C for 30min for colour development, and absorbance read at 765nm using a spectrophotometer. The total phenolic content was then expressed as mg/g gallic acid (GAE/gm) equivalent using the following equation based on the calibration curve:

$$Y\;=0.0052x;\;R^{2}=0.9846$$

#### Estimation of total flavonoid content

The total flavonoid content was estimated spectrophotometrically by using the aluminium chloride colourimetric assay[28]. The solution was made up of 0.5ml of the plant extract, 2ml of distilled water in a tube and 0.15 ml of 5% sodium nitrite. The solution was left for 5 min at room temperature then 0.15 ml of 10% aluminium chloride was added it and incubated for another 5 min. After incubation, 1ml of 4% sodium hydroxide was added and the solution made up to 5 ml with distilled water. It was then vortexed and incubated for 15 min to observe a colour change. Absorbance was measured at 420nm. The total flavonoid content was calculated as mg/g quercetin equivalent using the following equation from the calibration curve:

$$Y\;=0.0029x;\;R^{2}=0.997$$

#### Estimation of total flavonol content

The total flavonol content was estimated using the method described by Wintola and Afolayan [29]. 2 ml of the plant extract was added to 2 ml of 10% aluminium chloride prepared in ethanol. To this 3 ml of 5%, sodium acetate was added and then incubated at 20°C for 2^1^/_2_ h. The absorbance was measured at 440nm with a spectrophotometer. Total flavonol content was expressed as mg/g of quercetin equivalent derived from the following calibration curve:

$$Y\;=0.0107x;\;R^{2}=0.9928$$

#### Estimation of proanthocyanidin

The total proanthocyanidins were estimated using the method described by Caceres- Mella *et al*. [30]. 0.5 ml of the prepared plant extract was added to 3 ml of 4% vanillin-methanol. 1.5 ml of hydrochloric acid was then added to the solution and vortexed. The solution was left for 15 min at room temperature. The absorbance was then read at 500 nm using a spectrophotometer. Total proanthocyanidins content was calculated as mg/g of catechin equivalents using the equation derived from the calibration curve:

$$Y\;=0.0025x;\;R^{2}=0.9923$$

#### Estimation of tannin content

The total tannin content was estimated using the Folin—Ciocalteu method[28]. 7.5 ml of distilled water was added to a tube containing 0.1ml of the plant extract. 0.5 ml of Folin-Ciocalteuphenol reagent and 1 ml of 35% Na_2_CO_3_ solution was then added. The whole solution was made up to 10 ml with distilled water. The mixture was vortexed and kept at room temperature for 30 min. The absorbance was read at 725 nm using a spectrophotometer. A prepared set of standards of gallic acid was prepared in the same manner as the extracts as described earlier. The total tannin content was expressed as mg/g GAE using the following equation derived from the calibration curve:

$$Y\;=0.0122x;\;R^{2}=0.9838$$

#### Estimation of alkaloids content

The total alkaloid content was estimated using the method described by Unuofin *et al*.[31]. 5 g of the pulverized plant was soaked in 200 mL of 10% acetic acid in ethanol. It was allowed to stand for 4h at room temperature. It was subsequently filtered, and the filtrate was concentrated using a water bath at 55°C to a quarter of its original volume. Concentrated ammonium hydroxide was added in single drops until completion of the precipitation process. The solution was then washed with dilute ammonium hydroxide and filtered again. The residue obtained was first dried and then weighed. The alkaloid content was calculated using the equation:

$$\%\;Alkaloid=\;\frac{Weight\;of\;precipitate}{Weight\;of\;original\;sample}\;\times 100$$

#### Estimation of saponin

The saponin content was determined according to the method described by Omoruyi *et al*. [32] with some modifications. 5 g of the pulverized plant was added to 20 ml of 20% ethanol and extracted on a shaker for 30 min. The plant sample was heated over a water bath at 55°C for four h. The mixture was filtered, and the residue was re-extracted again with 20 ml of 20% aqueous ethanol. The filtrate was then reduced to 40 ml over a water bath at 90°C. The concentrate was transferred into a 250 ml separatory funnel, and extracted twice with 20 ml diethyl ether. The ether layer was discarded while the purification process was repeated. Sixty millilitres (60 ml) of n-butanol was added, and the extract was washed twice with 10 ml of 5% aqueous sodium chloride. The remaining solution was heated over a water bath and evaporated to dryness to a constant at 40°C. The saponin content was calculated using the following equation:

$$\%\;Saponin\;content\;=\;\frac{Weight\;of\;residue}{Weight\;of\;sample}\;\times 100$$

#### Estimation of phytate content

The total phytate content was estimated using the method described by Unuofin *et al*. [31]. 2 g of the pulverized plant was soaked into a conical flask with 50 ml of 2% hydrochloric acid for 3h and afterwards filtered. 25 ml of the filtrate was taken, and 5 ml of 0.3% ammonium thiocyanate solution was added. 53.5 ml of distilled water was also added to achieve the desired acidity. Then 0.05 M of iron III chloride was titrated into it until a reddish brown colour persists for 5 min. Phytate content was calculated as:

Phytate(%)=titrevalue×0.00195×1.19×100

### Antioxidant assays

#### DPPH radical scavenging assay

For DPPH radical scavenging activity of the plant extracts, the method described by Olajuyigbe and Afolayan [33] was adopted with some modifications. 1 ml of 0.135 mM DPPH in methanol solution was put into tubes with 1ml of various concentrations (0.2–1.0 mg/ml) of the plant extracts, vitamin C, and gallic acid. The mixture was vortexed, then left in the dark at room temperature for 30 min. The absorbance of the mixture was then measured spectrophotometrically at 517 nm. Both vitamin C and gallic acid were used as standards. The DPPH radical scavenging activity was calculated from the equation:

$$DPPH\;radical\;scavenging\;activity\;=\;\frac{Abs\;control-Abs\;sample}{Abs\;control}\;\times 100$$

where Abs control was the absorbance of DPPH radical + methanol; Abs sample was the absorbance of DPPH radical + sample extract or standards (Vitamin C and gallic acid).

#### Nitric oxide scavenging activity

Nitric oxide scavenging activity was determined according to the method described by Boora *et al*.[34] with some modifications. 2 ml of 10 mM Sodium nitroprusside was prepared in 0.5 ml phosphate buffer saline (pH 7.4) and mixed with 0.5 ml of either plant extracts, vitamin C or gallic acid, at various concentrations (0.2–1.0 mg/ml). The mixture was incubated at 25°C for 150 min. After incubation, 0.5 ml of Griess reagent (1.0 ml of 0.33% sulfanilic acid reagent prepared in 20% glacial acetic acid at room temperature for 5 min with 1 ml of naphthylethylenediamine dichloride) was added to an equal volume of the incubated solution. The mixture was incubated for another 30 min at room temperature, and the absorbance was then measured at 540 nm. The amount of nitric oxide radical inhibited by the extracts was calculated using the following equation:

$$NO\;radical\;scavenging\;activity\;=\;\frac{Abs\;control-Abs\;sample}{Abs\;control}\;\times 100$$

where Abs control was the absorbance of NO radical + methanol; Abs sample was the absorbance of NO radical + sample extract or standards (Vitamin C and gallic acid).

#### Hydrogen peroxide radical scavenging assay

For Hydrogen peroxide scavenging activity of the extracts, it was determined using the method described by Oyedemi *et al*. [35]. 4 ml of plant extract, vitamin C or gallic acid was prepared in distilled water at different concentrations (0.2–1.0 mg/ml) and mixed with 0.6 ml of 4 mM Hydrogen peroxide (H_2_O_2_) solution prepared in phosphate buffer (0.1 M, pH 7.4). The solution was incubated for 10 min at room temperature. The absorbance of the solution was then measured at 230 nm. The amount of hydrogen peroxide radical inhibited by the extract was calculated using the following equation:

$$H_{2}O_{2}\;radical\;scavenging\;activity\;=\;\frac{Abs\;control-Abs\;sample}{Abs\;control}\times 100$$

where Abs control was the absorbance of H_2_O_2_ radical + methanol; Abs sample was the absorbance of the H_2_O_2_ radical + sample extract or standard (Vitamin C and gallic acid).

#### Phosphomolybdenum antioxidant assay

The method adopted by Ahmed *et al*. [36] was used to determine the total antioxidant capacity with some modifications. 0.5 ml of plant extracts, vitamin C and gallic acid prepared in varying concentrations (0.1–0.5 mg/ml) were mixed with three ml of distilled water and 1ml of phosphomolybdate reagent in test tubes. The solutions were put in an incubator at 95°C for 90 min. After incubation, the tubes were normalized to room temperature for about 30min. Absorbance was measured at 695 nm. The amount of phosphomolybdenate inhibited by the extract was calculated using the following equation:

$$Phosphomolybdate\;antioxidant\;activity\;\;=\;\frac{Abs\;control-Abs\;sample}{Abs\;control}\times 100$$

where Abs control was the absorbance of phosphomolybdate reagent + methanol; Abs sample was the absorbance of phosphomolybdate reagent + sample extract or standard (Vitamin C and gallic acid).

### Vitamins estimation

#### Vitamin A estimation

Vitamin A estimation was done by the method described by Onyesife *et al*. [37]. 20ml of petroleum ether was added to 1g of pulverized plant and put on a shaker for about 30mins. The petroleum ether was decanted and evaporated to dryness. 0.2ml of chloroform-acetic anhydride (1:1 v/v) was added to the residue. Later on 2ml of trichloroacetic acid- chloroform (1:1 v/v) was added. The absorbance of the solution was then measured at 620 nm. The vitamin A standard was also prepared in the same way at varying concentrations, and a standard curve plotted. Results were expressed in mg/100g and calculated from the following equation based on the calibration curve:

$$Y\;=0.001x\;+0.0018;\;R^{2}=0.9922$$

#### Vitamin C estimation

Vitamin C estimation was done by the method described by Igwe and Okwu [38]. A 1g of the pulverised plant was put in 20ml of 0.4% oxalic acid. It was then filtered using a Whatman filter paper, and 1ml of the filtrate was mixed with 9ml of indophenol reagent. The absorbance of the solution was measured at 520nm. The vitamin C standard was also prepared in the same way at varying concentrations, and a standard curve plotted. Results were expressed in mg/100g using the following equation based on the calibration curve:

$$Y\;=0.67x\;+0.0824;\;R^{2}=0.9714$$

#### Vitamin E estimation

Vitamin E estimation was done by the method described by Onyesife *et al*.[37]. 20ml of ethanol was added to 0.5g of the pulverized sample and then left on a shaker for 20mins. It was then filtered. 1ml of the filtrate was then mixed with 1ml of 0.2% of ferric acid in ethanol and 1ml of 0.5% α-α-dipyridine. The solution was made up to 5mls with distilled water. The absorbance of the solution was read at 520 nm. The vitamin E standard was also prepared in the same way at varying concentrations, and a standard curve plotted. Results were expressed in mg/100g using the following equation based on the calibration curve:

$$Y\;=0.0086x\;+0.0216;\;R^{2}=0.99$$

### Determination of anti-inflammatory activity (Cell line and cell culture)

The RAW 264.7 cells were obtained from the cell culture lab at the Department of Biochemistry, Nelson Mandela University. The RAW 264.7 cells were first suspended in Dulbecco’s Modified Eagle Medium (DMEM/low Glucose solution) (Hyclone Laboratories, U.S.A) supplemented with 10% heat-inactivated fetal bovine serum (FBS) and antibiotics (100 U/mL penicillin and 100 U/mL streptomycin) at 37°C. The cells were stained with trypan blue and the number of viable cells counted using an inverted light microscope (Zeiss) and a Neubauer counting chamber (Hausser Scientific, USA Sigma Cat Z359629).The RAW 264.7 cells were then put into the incubator at 37°C for 24h under a humidified atmosphere of 5% CO2 to allow for acclimatization.

#### Quantification of nitric oxide production

After 24h (the cells had adhered to the surface), 100μl of the cells each were taken and put in separate wells in a 96 well culture plate. Fifty microliters (50μl) of Lipopolysaccharide (LPS), at either 100μM or 25μM, was added to each well together with 50μl of plant extracts (25 and 100μg/ml). The 96-well plates were then put back in the incubator at 37 ^o^C for 24hrs. Aliquots (50μl) of the cells from each well were removed and added to 50μl of Griess reagent (Sigma Cat# 03553) in another 96-well culture plate and incubated at room temperature for 15 min. The absorbance was read at 560nm in a microplate reader (Multiscan MS, Labsystems). All tests were done in triplicate.

#### Methyl-thiazolyl tetrazolium (MTT) assay

The cytotoxicity assay was carried out on the LPS -induced cells and those with plant extracts and controls using the MTT assay^13^. One hundred microliters (100μl) of RAW 264.7 cells were aliquoted into wells of a 96-well culture plate, and One hundred microliters (100μl) of 3-(4,5-dimethylthiazol-2-yl)-2,5-diphenyltetrazolium bromide added to each well and incubated at room temperature for 1h. The cell viability was measured at an optical density of 560 nm in a microplate reader (Multiscan MS, Labsystems) All tests were done in triplicate.

#### Cyclo-oxygenase 2 (COX-2) assay

The RAW 264.7 cells were fixed in formaldehyde at two different concentrations (100μM and 25μM). The formaldehyde was removed by solubilizing in methanol (50μl), and the remaining RAW 264.7 cells kept in the freezer at -20°C for 10 min. The cells were then washed with 50μl of 1% bovine serum albumin (BSA) solution, and 50μl of 3% BSA and 0.2% Triton X100 added before incubating at room temperature for 45min. Fifty microliters (50μl) of antibody was added at 1: 800 dilution and the cells incubated for a further 1h. The cells were then washed twice with PBS before Hoechst solution with 3% BSA was added. The presence of the COX-2 was observed using a fluorescent microscope (ImageXpress XLS Micro [Molecular Devices]). All tests were done in triplicates.

#### Determination of cytotoxicity

Fresh and dried plant extracts of the various solvents (aqueous, acetone & ethanol) were weighed and dissolved in dimethyl sulfoxide (DMSO). They were then all sonicated for proper solubilization and stored at -20°C. The plant extracts were then diluted in RPMI-1640 supplemented with 5% fetal bovine serum (FBS) and streptomycin medium to its final concentrations.

The U937 and Jurkat cell lines used were obtained from the Department of Biochemistry, Nelson Mandela University, South Africa. The cell lines were maintained in RPMI-1640 (Hyclone Labs, U.S.A) medium supplemented with 5% FBS and penicillin/streptomycin (Biowest, U.S.A). The cells were plated at a density of 2.5 x 10^4^ cells/well in a 96-well plate and cultured overnight in an incubator at 37°C. After 24h the plant extracts were added at various concentrations (12.5,25,50, 100, and 200 μg/ml for U937; 25,50, 100 and 200 μg/ml for Jurkat), then the cells were incubated for another 48h. MTT assay was performed as previously described and the plates incubated for 4h. The absorbance was determined using an ELISA reader (Multiscan MS, Labsystems) at a wavelength of 560nm. All the tests were done in triplicate. Cytosine arabinoside was used as a positive control. Cell viability of the treated cells was determined in reference to the untreated control cells using the following formula:

$$\%\;Cell\;viability=\;\frac{Sample\;Abs}{Control\;Abs}\;\times 100$$

### Gas chromatography-mass spectroscopy (GC-MS) analysis

The GC-MS analysis was performed to determine the chemical make-up of Opuntia stricta cladodes. Fresh Opuntia cladodes were first extracted of volatile oil using a hydro-distiller in a Clevenger’s-type apparatus in accordance with the British Pharmacopeia specifications (1980). The analysis was performed using Agilent 6890 GC coupled to Agilent 5975 MSD with a Zebron-5MS column (ZB-5MS 30 m x 0.25 mm x 0.25 um). GC grade helium was used as a carrier gas at a flow rate of 2 mL/min; splitless one μL injections were used. The temperature of the injector was 280°C; the source 280°C, the oven 70°C, the ramp was 15°C/min. to 120°C, then 10°C/min to 180°C, then 20°C/min. to 270°C and held for 3 minutes. The compounds present in the essential oils were identified by matching their spectral mass against the National Institute of Standards and Technology (NIST) 11 database.

### Statistical analysis

Data obtained were presented as means ± SD. All experiments were done in triplicates. One way analysis of variance (ANOVA) and the Tukey test were used to determine the differences among the means of the various samples. P values < 0.05 were regarded to be significant.

## Results

### Phytochemical composition

**Fig 1:**
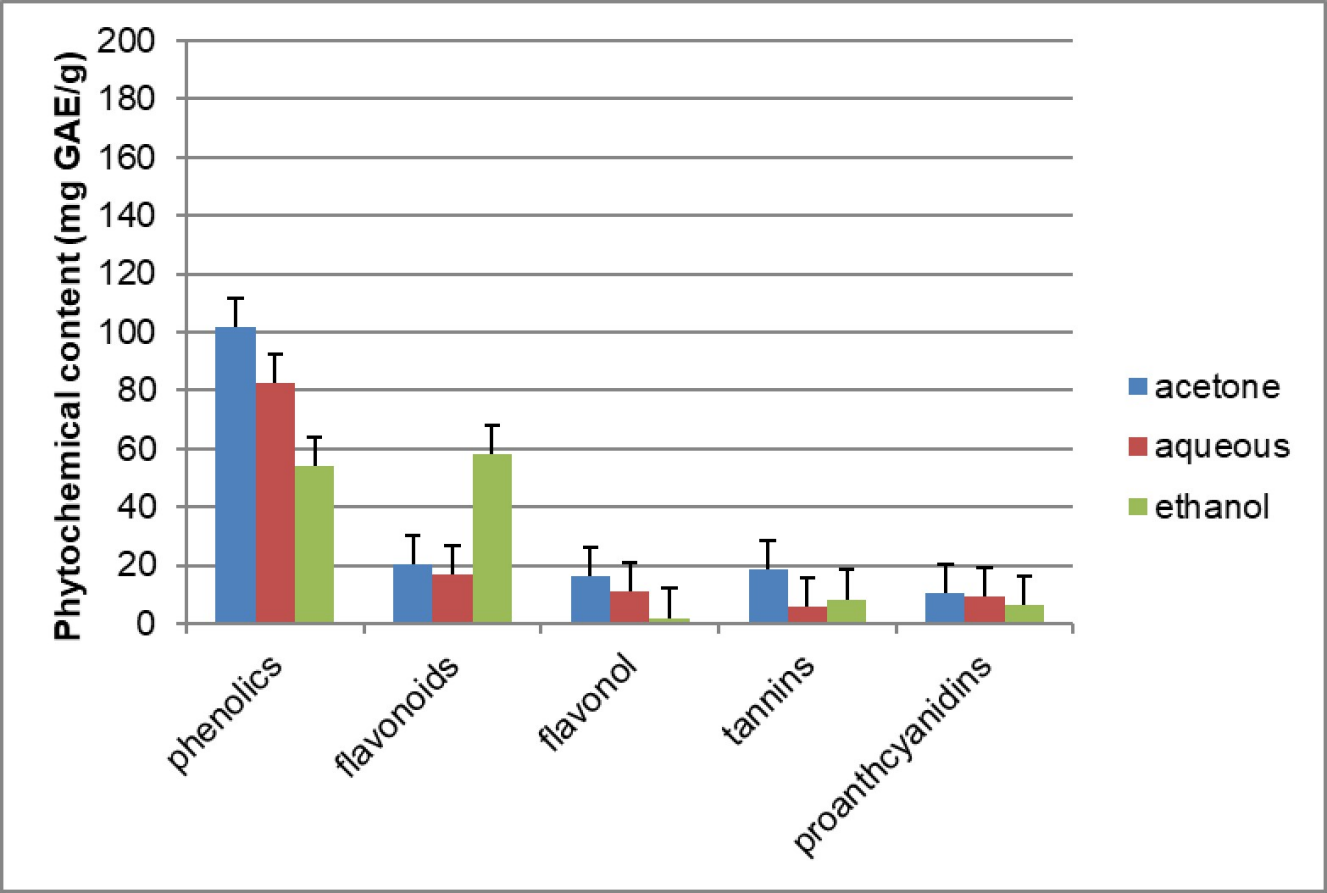
Phytochemical constituents identified in the various extracts of *Opuntia stricta*. Values are expressed as mean ± standard deviation (SD) of three separate determinations (n = 3).

In this study, the amount of the various phytochemicals investigated varied significantly among the various extracts studied (Fig 1). The total phenolic content of the different solvent extracts of *Opuntia stricta* cladodes showed variable yields in this study. The yields were 101.81mg/g, 82.54mg/g, 54.98mg of GAE equivalent per gram of dried extract of acetone, aqueous and ethanol respectively. The acetone extract of the *Opuntia stricta* cladodes yielded significantly higher phenolic contents than the aqueous and ethanol extract (P < 0.05).The aqueous extract was also significantly higher than the ethanol extract (P< 0.05). The total flavonoid content of the ethanol extract of *Opuntia stricta* exhibited a higher yield of 57.93mg of quercetin equivalent per gram of dried extract. The acetone and aqueous yields were 20.37, 17.01 mg of quercetin equivalent per gram of dried extract respectively. The ethanol extract showed significantly higher yields than the aqueous or acetone extract (P< 0.05). The acetone extract had a higher flavonol content at 16.11mg of quercetin equivalent per gram of dried extract compared to aqueous extract of 10.84mg of quercetin equivalent per gram of dried extract. The ethanol extract was negligible at 1.9mg of quercetin equivalent per gram of dried extract. The acetone and the aqueous extracts showed significantly higher yields when compared to the ethanol extract (P<0.05). The acetone extract of *Opuntia stricta* cladodes gave the highest yield of total proanthocyanidin content at 10.4mg of catechin equivalent per gram of dried extract. The catechin equivalent per gram of dried extract of the aqueous and ethanol extracts were 9.2 and 6.4mg, respectively. The total tannin content of the acetone, aqueous and ethanol extracts were 18.38,5.84 and 8.32mg of GAE equivalent per gram of dried extract, respectively.

The anti-nutrient content alkaloid, saponin and phytate of the dry macerated cladodes are listed in Table 1. The saponin content was much higher than the phytate and alkaloid.

**Table 1:** Anti-nutrient composition of *Opuntia stricta* cladodes.

Bioactive compound	Content (%)
Saponin	93.8 ± 3.43
Alkaloid	0.32 ± 0.02
Phytate	0.37 ± 0

### Vitamin contents of *Opuntia stricta*

Vitamins A, E, and C were present in the dried cladodes of *Opuntia stricta* (Table 2). The highest vitamin content was vitamin A at 711.2 mg/100g of dried extract. The vitamin E content was 231.4 mg /100g of dried extract. The vitamin C content was the least of all the measured vitamins at 2.9mg /100g of dried extract.

**Table 2:** Vitamin content of *Opuntia stricta* cladodes (dried weight).

Vitamin	Mean ± SD (mg/100g)
Vitamin A	711.2 ± 4.2
Vitamin E	231.4 ± 18.6
Vitamin C	2.9 ± 0.08

### DPPH radical scavenging activity

**Fig 2:**
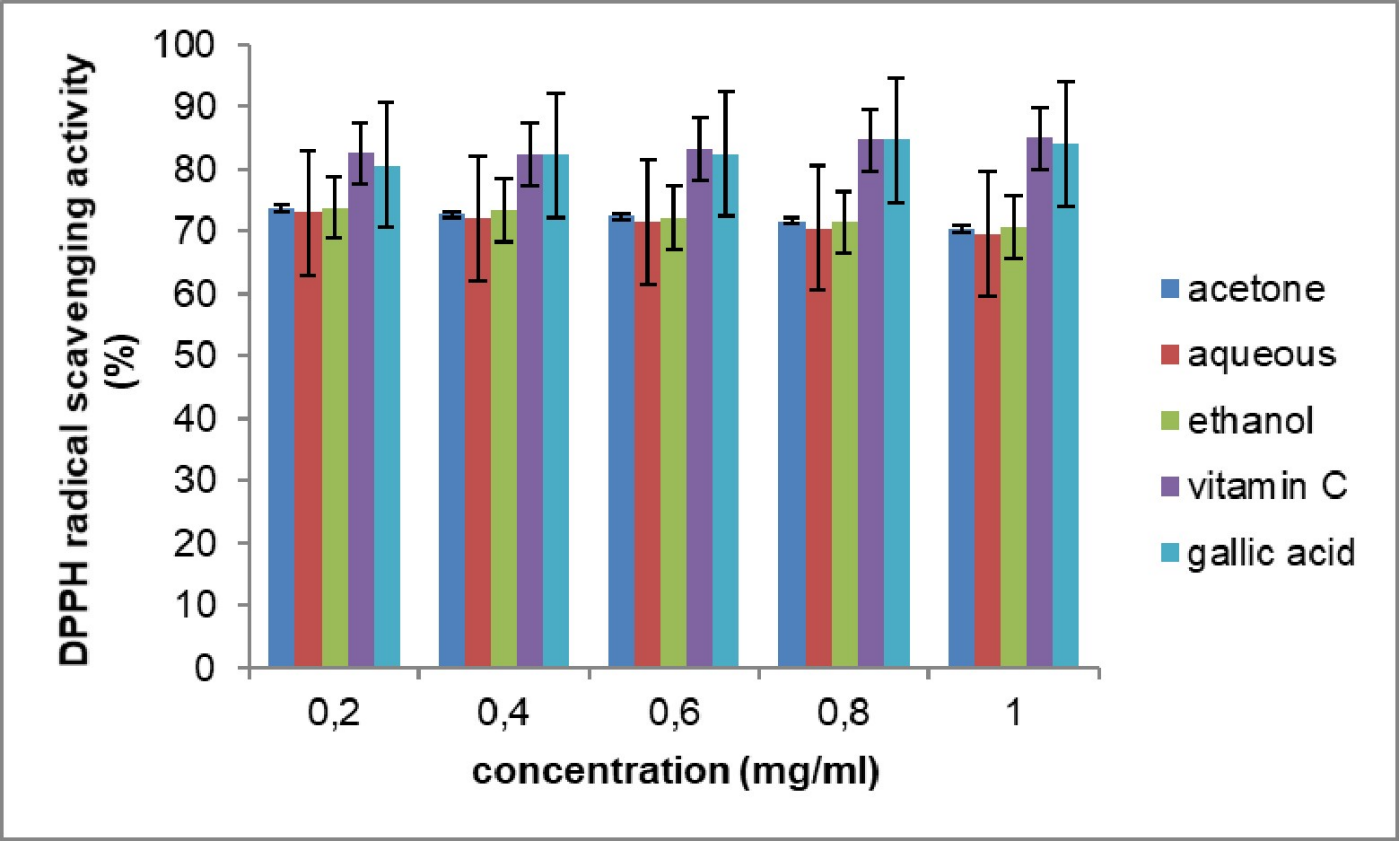
DPPH radical scavenging activity of the extracts of *Opuntia stricta* cladodes. The values represent mean ± S.D (n = 3).

The results of the DPPH scavenging activity of the extracts are as shown in Fig 2. The results showed that ethanol extract had the highest scavenging activity of the extracts at 73.79% ± 0.01. Although none of the extracts in this study had a higher activity than vitamin C and gallic acid used as standards. The acetone, aqueous, ethanol, vitamin C and gallic acid had IC50 values of 0.511, 0.518, 0.510, 0.436 and 0.439mg/, respectively. The study showed that the various extracts have compounds capable of donating protons to the free radicals. This confirms the ability of *Opuntia stricta* to exhibit DPPH free radical scavenging activity.

### Nitric oxide scavenging activity

**Fig 3:**
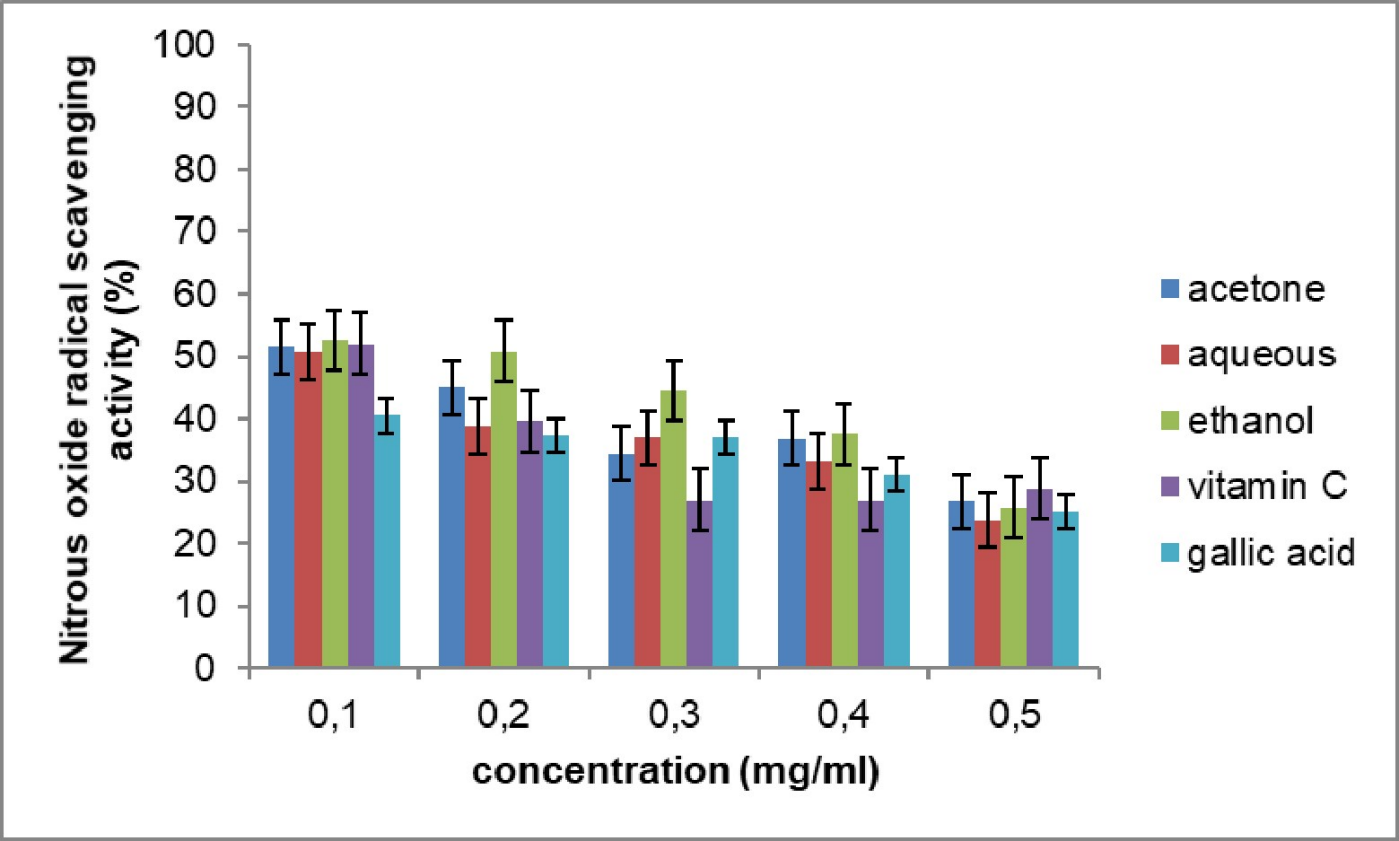
Nitric oxide radical scavenging activity of the extracts of *Opuntia stricta* cladodes. The values represent mean ± S.D (n = 3).

In this study, the extracts and standards (vitamin C and gallic acid) show a concentration- dependent scavenging activity. The ethanol extract showed the highest antioxidant activity at 52.54% ± 0.1. It was higher than the standards used in this study. The IC50 values of the various extracts (ethanol, 0.97mg/ml; acetone, 1.04mg/ml; aqueous, 1.12mg/ml) were comparable to vitamin C (1.18mg/ml) and gallic acid (1.18mg/ml). The result of the nitric oxide scavenging activity is depicted in Fig 3.

### Hydrogen peroxide scavenging activity

**Fig 4:**
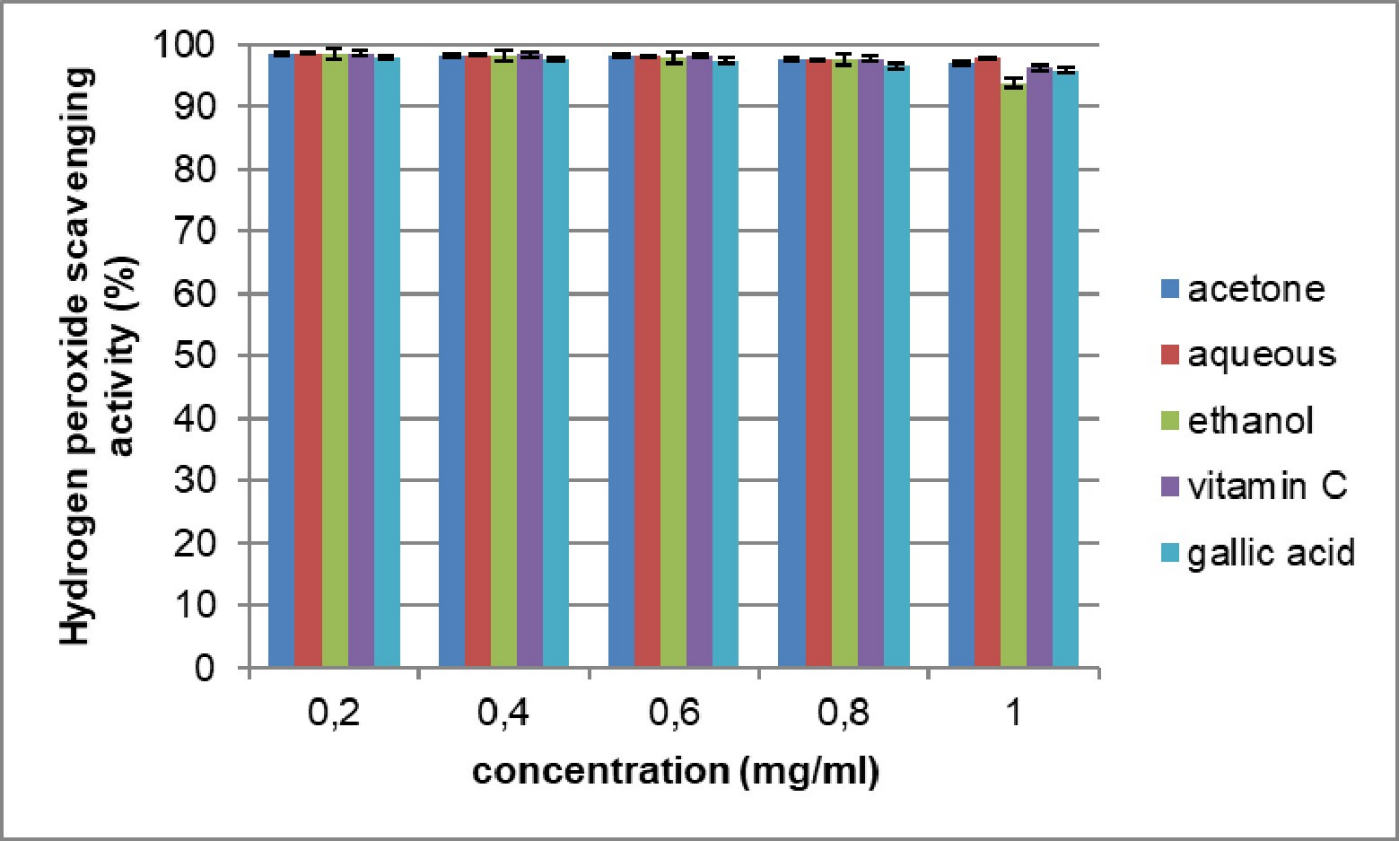
Hydrogen peroxide scavenging activity of the extracts of *Opuntia stricta* cladodes. The values represent mean ± S.D (n = 3).

In this study, the extracts and standards (vitamin C and gallic acid) show a concentration- dependent scavenging activity. The result showed that the aqueous extract had the highest scavenging activity at 98.63% ± 0.01. It was equally higher than the standards used. All the extracts and standards used recorded high inhibitory activities. The acetone, aqueous, ethanol, vitamin C and gallic acid had IC50 values of 0.375, 0.374, 0.379, 0.376 and 0.379mg/ml respectively. These results show that all the extracts used are capable of inhibiting hydrogen peroxide radical. Fig 4 shows the results of hydrogen peroxide scavenging activity.

### Phosphomolybdenum (total) scavenging activity

**Fig 5:**
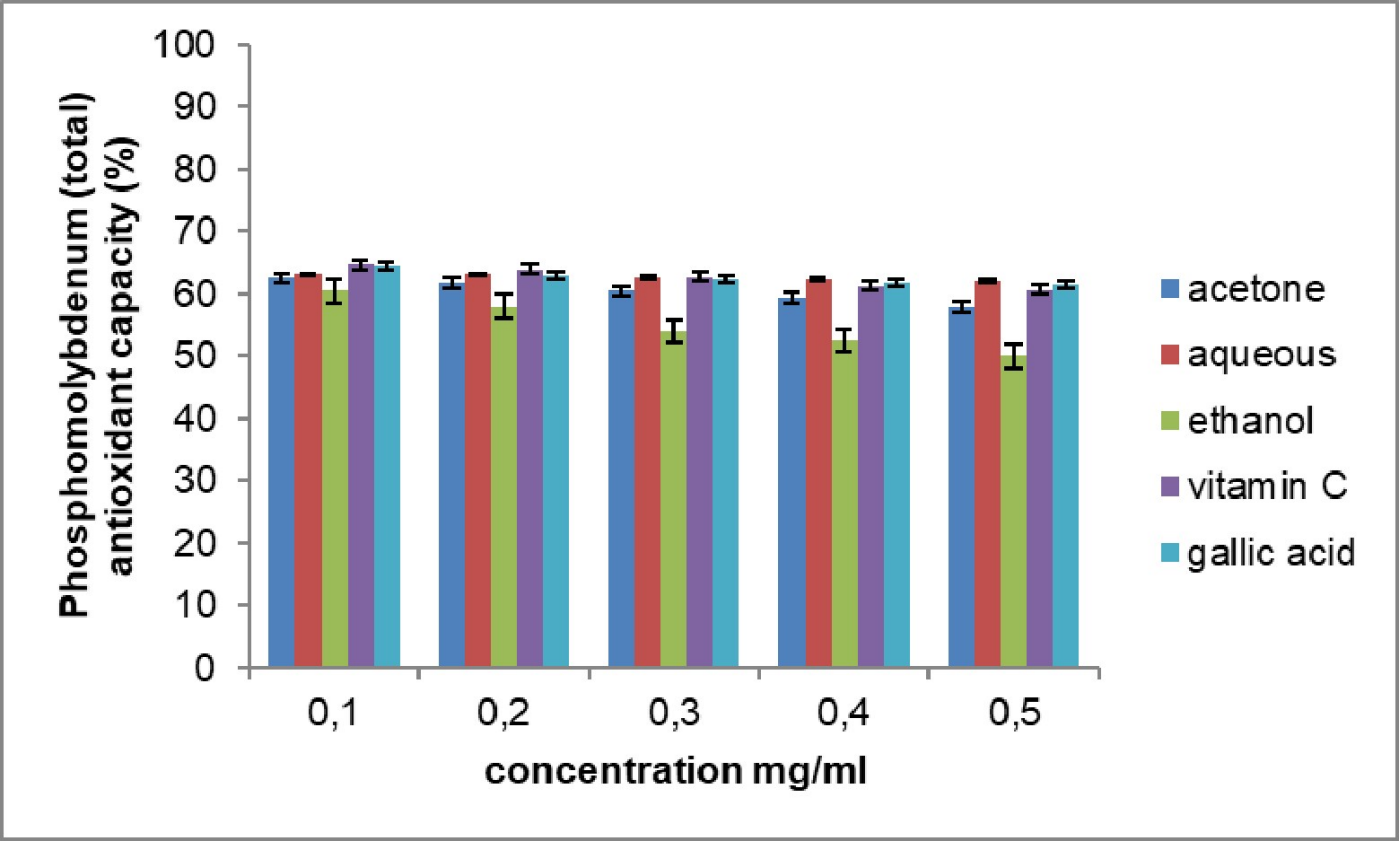
Phosphomolybdenum (total) antioxidant activity of the extracts of *Opuntia stricta* cladodes. The values represent mean ± S.D (n = 3).

The reducing abilities of the different extracts determined by Phosphomolybdenum (total) scavenging activity were measured spectrophotometrically by their absorbances and summarized in Fig 5. The extracts and standards (vitamin C and gallic acid) show a concentration- dependent scavenging activity. In this study, the aqueous extract of *Opuntia stricta* exhibited the highest total antioxidant capacity at 67.87% ± 0.004. The acetone and the ethanol extracts had activities of 66.15% ± 0.006 and 65.97% ± 0.001 respectively. The scavenging activity of the three extracts was not significantly different and was higher than that for vitamin C and gallic acid. The IC50 values of 0.297, 0.3158, 0.2959, 0.2961, 0.2952 mg/ml were recorded for the acetone, aqueous, ethanol, vitamin C and gallic acid respectively. This study confirms the antioxidant capacity of *Opuntia stricta* cladodes, and its ability to mop up free radicals.

### GC-MS analysis of the essential oils

**Fig 6:**
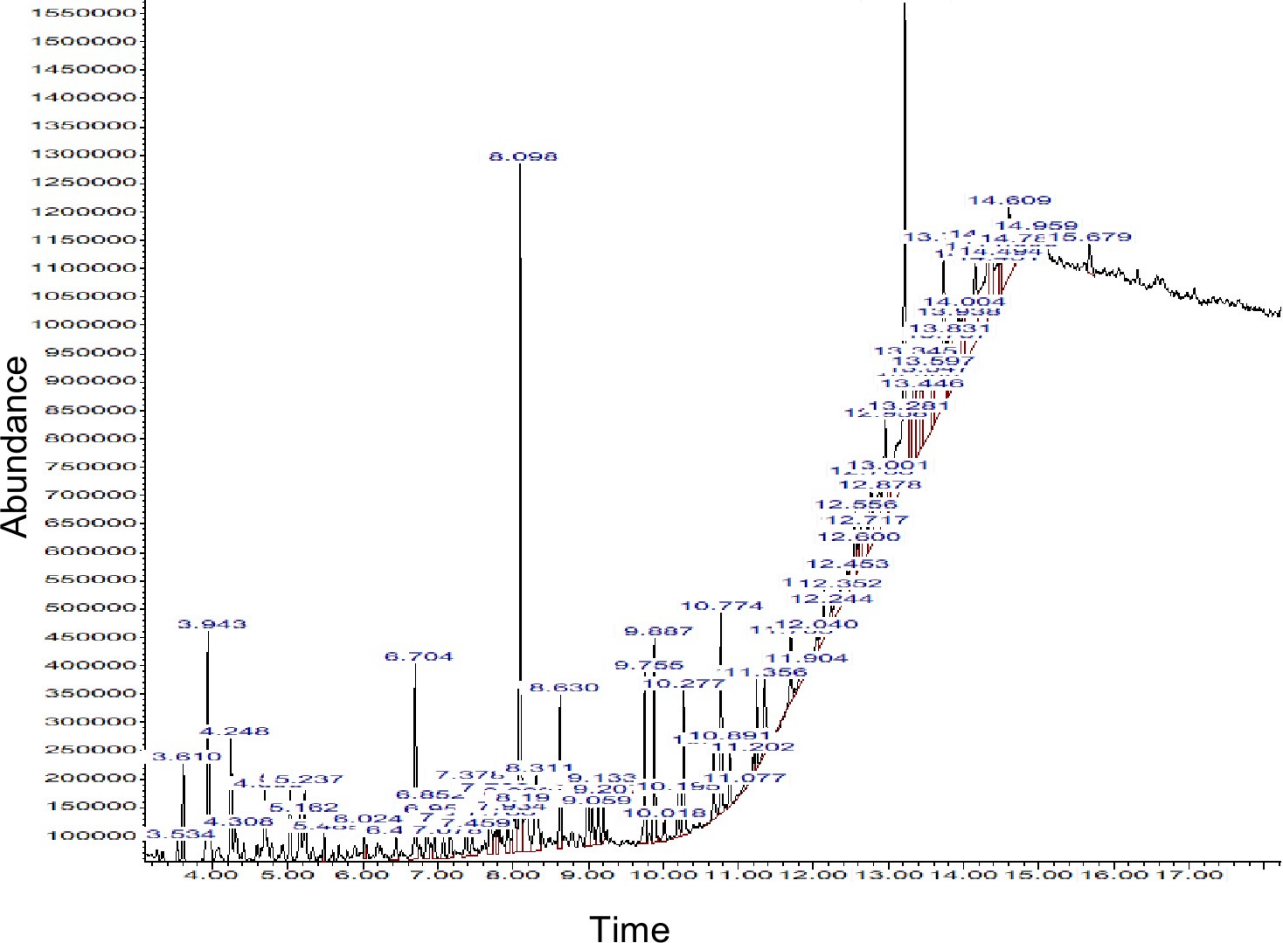
GC-MS chromatogram of essential oil of *Opuntia stricta*.

The GC-MS analysis was carried out in order to discover any antioxidant, anti-inflammatory or cytotoxic compound in the essential oil (Fig 6). 46 compounds were identified in the essential oils of *Opuntia stricta*. They include beta-copaene (7.54%), octasiloxane 1,1,3,3,5,5,9,9, 11,11,13,13,15,15- hexadecamethyl- (4.75%), Cyclotrisiloxane, hexamethyl- (3.83%), alpha-pinene (3.12%), alpha.-Ionone (2.45%), and 5-Methyl-2-trimethylsilyloxy-acetophenone (2.33%). The composition of compounds in the essential oil consisted of monoterpenes, cyclic terpenes, sesquiterpenes, fatty acids, phenols, alcohols, and aromatic compounds. Some of these compounds are known to exhibit some biochemical activity. Beta-ionone and terpinolene, two of the compounds found in the essential oil of *Opuntia stricta* are known to exhibit antioxidant, anti-inflammatory and cytotoxic activities[39][40]. The various compounds found in the essential oils of the plant are listed in Table 3.

**Table 3:** Chemical composition of *O*. *stricta* essential oil determined by GC-MS.

S/N	RT (mins)	Compounds	Peak (%)	Structure (ChemSpider Link Address)	Molecular formula	Function	MW (Da)	Hit quality
1	3.534	Dodecamethylcyclohexasiloxane	0.3	http://www.chemspider.com/Search.aspx?rid=497f293c-cce4-45e3-a89e-f37191f1fa83&seq=20	C_12_H_36_O_6_Si_6_		444.924	46
2	3.613	Heptanal	1.23	http://www.chemspider.com/Search.aspx?rid=b745fc96-2f9b-44a1-8c0e-c14fd07a0193&seq=20	C_7_H_14_O		114.186	97
3	3.613	Hexanal, 3-methyl-	1.23	http://www.chemspider.com/Search.aspx?rid=ef463d21-63b9-44a0-9283-71022c48f8eb&seq=20	C_7_H_14_O		114.186	37
4	3.613	Formamide, N,N-dimethyl-	3.12	http://www.chemspider.com/Search.aspx?rid=84c2fdd6-d38b-4079-b6d2-512fc9c6c043&seq=20	C_3_H_7_NO		73.094	43
5	3.943	alpha-Pinene	1.46	http://www.chemspider.com/Search.aspx?rid=f238c795-72db-4343-a601-8a1818e8afb9&seq=20	C_10_H_16_	Antineoplastic, anti-inflammatory. antioxidant [41][42]	136.234	95
6	4.242	beta-Phellandrene	0.92	http://www.chemspider.com/Search.aspx?rid=84d8f020-b4da-42fe-afc0-c5ae0a27665e&seq=20	C_10_H_16_	Antineoplastic, anti-inflammatory. antioxidant [43]	136.234	91
7	4.695	alpha ocimene	0.92	http://www.chemspider.com/Search.aspx?rid=1a45cb0c-56b8-4ada-98a4-3fe8049fb11f&seq=20	C_10_H_16_	Anti-inflammatory[44]	136.234	55
8	4.695	sylvestrene	0.95	http://www.chemspider.com/Search.aspx?rid=49a53514-6df3-41ed-aab9-75f92423eaba&seq=20	C_10_H_16_		136.234	60
9	5.034	Linalool oxide	1.05	http://www.chemspider.com/Search.aspx?rid=31eef633-5312-41f9-b38d-7f2bfaa9dcc1&seq=20	C_10_H_18_O_2_	Antineoplastic, anti-inflammatory. antioxidant[45][46]	170.249	59
10	5.237	Nonanal	1.05	http://www.chemspider.com/Search.aspx?rid=90308de3-f4ee-4b83-96e3-5a9b156a9c7d&seq=20	C_9_H_18_O		142.239	68
11	5.237	Cycloheptane	0.36	http://www.chemspider.com/Search.aspx?rid=91248521-ecfc-4a11-905c-0b51102f12ef&seq=20	C_7_H_14_		98.186	47
12	6.024	N-Nitroso-di-n-octylamine	2.45	http://www.chemspider.com/Search.aspx?rid=59ef48c4-c7d5-4633-8737-bae3be493ec8&seq=20	C_16_H_34_N_2_O		270.454	27
13	6.704	Terpinolene	2.45	http://www.chemspider.com/Search.aspx?rid=cfe99125-e636-41e6-b8cb-06d15c9d2888&seq=20	C_10_H_16_	Antineoplastic, anti-inflammatory. antioxidant[47][48]	136.234	70
14	6.704	beta-Ionone	2.45	http://www.chemspider.com/Search.aspx?rid=6cd64782-03e4-407d-9b08-a00b092b4b45&seq=20	C_13_H_20_O	Antineoplastic, anti-inflammatory. antioxidant[49][50]	192.297	70
15	6.704	alpha-Ionone	0.83	http://www.chemspider.com/Search.aspx?rid=2ad01621-527a-4f6f-90d7-923007c087cd&seq=20	C_13_H_20_O		192.297	70
16	6.852	Theaspirane	0.52	http://www.chemspider.com/Search.aspx?rid=59ac34ee-ab5d-453e-80ba-664fa715f931&seq=20	C_13_H_22_O	Anti-inflammatory, antioxidant[51]	194.313	58
17	6.956	Trans-decalin	0.52	http://www.chemspider.com/Search.aspx?rid=a0ea4e92-5d47-46c2-af2e-4c38d5526956&seq=20	C_10_H_18_		138.250	50
18	6.956	Gephyrotoxin 181b	0.88	http://www.chemspider.com/Search.aspx?rid=134bd5ec-8d71-44e9-b9a6-48baca292695&seq=20	C_12_H_23_N		181.318	50
19	7.378	Octadecane, 1-iodo-	7.54	http://www.chemspider.com/Search.aspx?rid=2f126460-54fc-4fcf-8031-c08c5562161d&seq=20	C_18_H_37_I		380.391	25
20	8.098	beta-copaene	7.54	http://www.chemspider.com/Search.aspx?rid=f4e46c4e-552d-45c4-8031-be58bc9dfe5e&seq=20	C_15_H_24_	Antineoplastic, antioxidant[52][53]	204.351	93
21	8.098	Gemacrene D	1.5	http://www.chemspider.com/Search.aspx?rid=de69d2a8-b6a1-4586-83bf-de083aa67166&seq=20	C_15_H_24_	Antineoplastic, anti-inflammatory[54]	204.351	96
22	8.311	Beta Cadinene	1.5	http://www.chemspider.com/Search.aspx?rid=9a9e795b-23b7-4353-94ed-c66044c410a8&seq=20	C_15_H_24_	Antineoplastic, anti-inflammatory[55]	204.351	95
23	8.311	Delta Amorphene	1.5	http://www.chemspider.com/Search.aspx?rid=2e1b0f9d-66bc-438d-9a11-c5dfbbfa4bb2&seq=20	C_15_H_24_	Antineoplastic, anti-inflammatory[55]	204.351	91
24	8.63	Hexadecane	1.5	http://www.chemspider.com/Search.aspx?rid=cf8c6e05-9e1e-4f88-8d0a-da9d220d682f&seq=20	C_16_H_34_		226.441	99
25	8.63	Tetradecane	1.5	http://www.chemspider.com/Search.aspx?rid=db7d9dcb-0cbc-4ac8-a5f6-39439f5fd32c&seq=20	C_14_H_30_		198.388	96
26	8.63	Octadecane	0.86	http://www.chemspider.com/Search.aspx?rid=3afdc06b-0a6d-426a-94fc-7f57030273d4&seq=20	C_18_H_38_	Antineoplastic analogue[56]	254.494	94
27	8.993	Ledol	0.86	http://www.chemspider.com/Search.aspx?rid=a95ef13f-2597-4cb8-ac0e-cb1c06b2762c&seq=20	C_15_H_26_O	Anti-inflammatory, antioxidant[57]	222.366	42
28	8.993	Guaia-3,9-diene	0.86	http://www.chemspider.com/Search.aspx?rid=5508bd39-9781-47a0-82d0-f1776e9e42bb&seq=20	C_15_H_24_		204.351	38
29	8.993	(-)-Globulol	0.68	http://www.chemspider.com/Search.aspx?rid=76b9c9cb-2f23-4140-b24f-06e03bcaf3a7&seq=20	C_15_H_26_O	Anti-inflammatory, antioxidant[58]	222.366	30
30	9.133	alpha-Cadinol	0.68	http://www.chemspider.com/Search.aspx?rid=12364970-0b3e-4548-bcab-3e1e03659c48&seq=20	C_15_H_26_O	Antioxidant[59]	222.366	94
31	9.133	Alloisolongifolene	0.38	http://www.chemspider.com/Search.aspx?rid=261a2d49-03f8-4617-a667-099d7a6522d2&seq=20	C_15_H_24_		204.351	46
32	9.207	Heptadecane	1.74	http://www.chemspider.com/Search.aspx?rid=8685f550-c262-41ff-a429-0280ed4b2c39&seq=20	C_17_H_36_	Anti-inflammatory, antioxidant[60]	240.468	95
33	9.755	Heneicosane	1.96	http://www.chemspider.com/Search.aspx?rid=dee77e36-a923-4641-b0c3-f69acc526437&seq=20	C_21_H_44_	Anti-inflammatory, antioxidant[61]	296.574	94
34	9.887	Isopropyl myristate	1.96	http://www.chemspider.com/Search.aspx?rid=c8ce7673-6f53-4573-8201-db6f22d4b1d6&seq=20	C_17_H_34_O_2_	Anti-inflammatory, antioxidant[61]	270.451	99
35	9.887	Palmitic acid	1.47	http://www.chemspider.com/Search.aspx?rid=a457ff5e-e9e6-4cb8-8240-399999b96f04&seq=20	C_16_H_32_O_2_		256.424	25
36	10.277	Nonadecane	1.47	http://www.chemspider.com/Search.aspx?rid=07227ed7-8df4-43e7-896f-015b0c505c34&seq=20	C_19_H_40_	Anti-inflammatory, antioxidant[61]	268.521	98
37	10.277	Eicosane	0.72	http://www.chemspider.com/Search.aspx?rid=d10b5c49-1ffe-4976-942e-0458389e99d6&seq=20	C_20_H_42_		282.547	96
38	10.678	Dibutyl phthalate	0.63	http://www.chemspider.com/Search.aspx?rid=9b254837-50a3-44dc-9359-a85b0645108e&seq=20	C_16_H_22_O_4_		278.344	92
39	10.891	Isopropyl palmitate	1.16	http://www.chemspider.com/Search.aspx?rid=58dd7f08-c63b-4406-9a00-10a4019b4c36&seq=20	C_19_H_38_O_2_	Anti-inflammatory, antioxidant[62]	298.504	46
40	11.356	Eicosane, 9-octyl-	0.93	http://www.chemspider.com/Search.aspx?rid=45e400f2-052d-4594-a1aa-4d8baf013d87&seq=20	C_28_H_58_		394.760	45
41	12.137	Hexamethylcyclotrisiloxane	0.72	http://www.chemspider.com/Search.aspx?rid=14038be8-653b-4c61-b3d9-2ee393afbeff&seq=20	C_6_H_18_O_3_Si_3_		222.462	43
42	12.717	2-Ethylacridine	9.14	http://www.chemspider.com/Search.aspx?rid=a2f71291-8f88-4aaf-ad0f-0e97fa30b993&seq=20	C_15_H_13_N		207.270	43
43	13.219	Diisooctyl phthalate	9.14	http://www.chemspider.com/Search.aspx?rid=a9059437-b847-4aba-b3d5-c12ac069018f&seq=20	C_24_H_38_O_4_		390.556	90
44	13.219	Bis(2-ethylhexyl) phthalate	2.33	http://www.chemspider.com/Search.aspx?rid=3dafedd2-7b2a-4f6b-be05-b86c664a6fc0&seq=20	C_24_H_38_O_4_	Pro-inflammatory[63]	390.556	60
45	13.547	5-methyl-2-trimethylsilyloxy-acetophenone	7.54	http://www.chemspider.com/Search.aspx?rid=2bd4fa4e-79d2-4fea-9edf-269a263c8792&seq=20	C_12_H_18_O_2_Si		222.356	38
46	8.098	beta Cubebene		http://www.chemspider.com/Search.aspx?rid=0166a07b-1a8c-447b-aa21-caadb983a5bf&seq=20	C_15_H_24_		204.351	94

### Effect of *Opuntia stricta* extracts on macrophage toxicity

**Fig 7:**
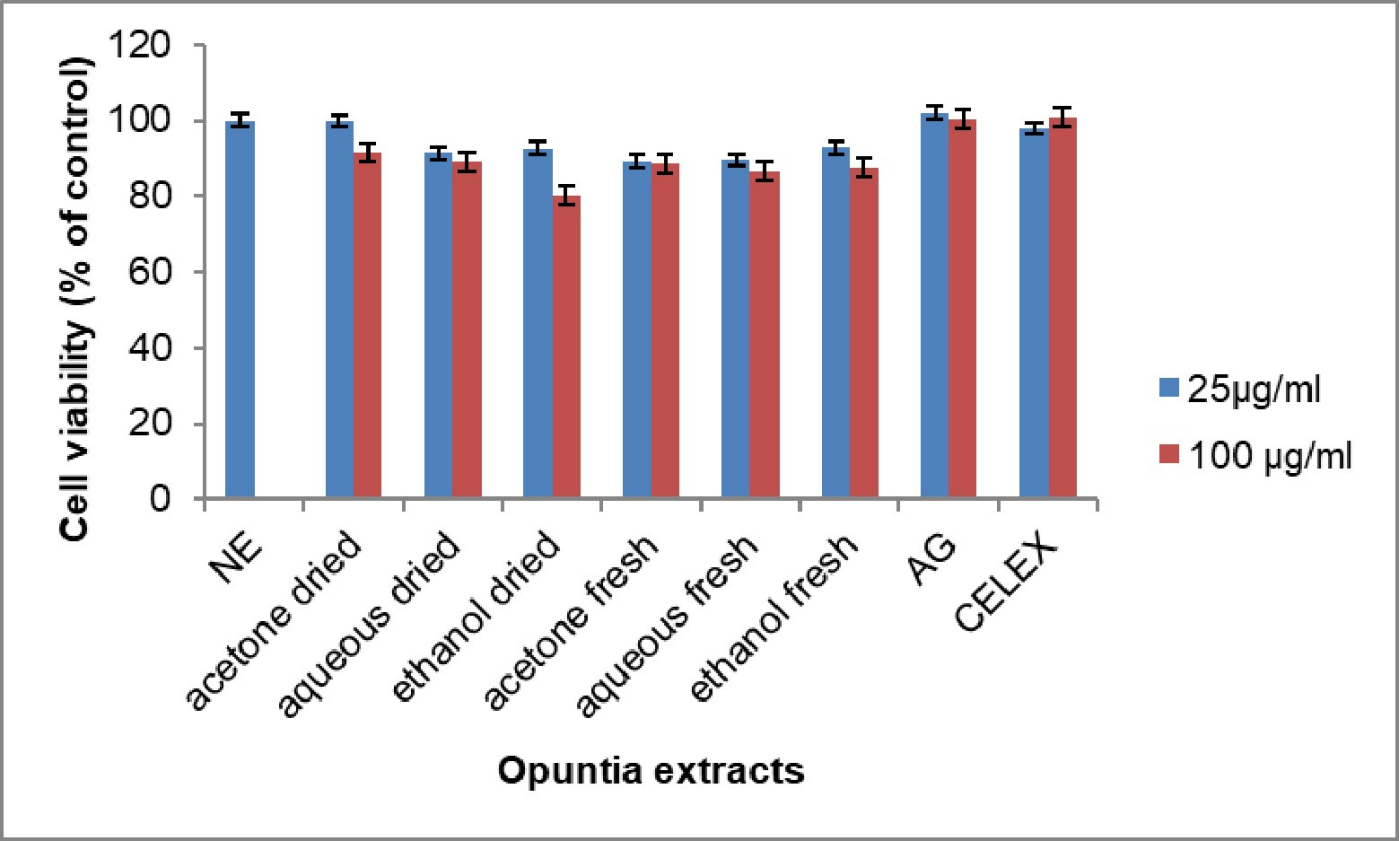
The percentage viable RAW 264.7 cells after 24-hour incubation with either 25 or 100µg/ml LPS and the *Opuntia* extracts. AG = Aminoguanidine. CELEX = Celecoxib. NE = No extract. Each value represents mean ± S.D (n = 3).

The RAW 264.7 cells were incubated with the plant extracts in two concentrations: 100μM and 25μM. Cell viability was measured by MTT assay as previously described. From the study, the Opuntia extracts had mild cytotoxic effects on the RAW 264.7 cells especially at 100 μM (Fig 7). These results proved that activities of Opuntia extracts were not as a result of a reduction in cell viability.

**Fig 8:**
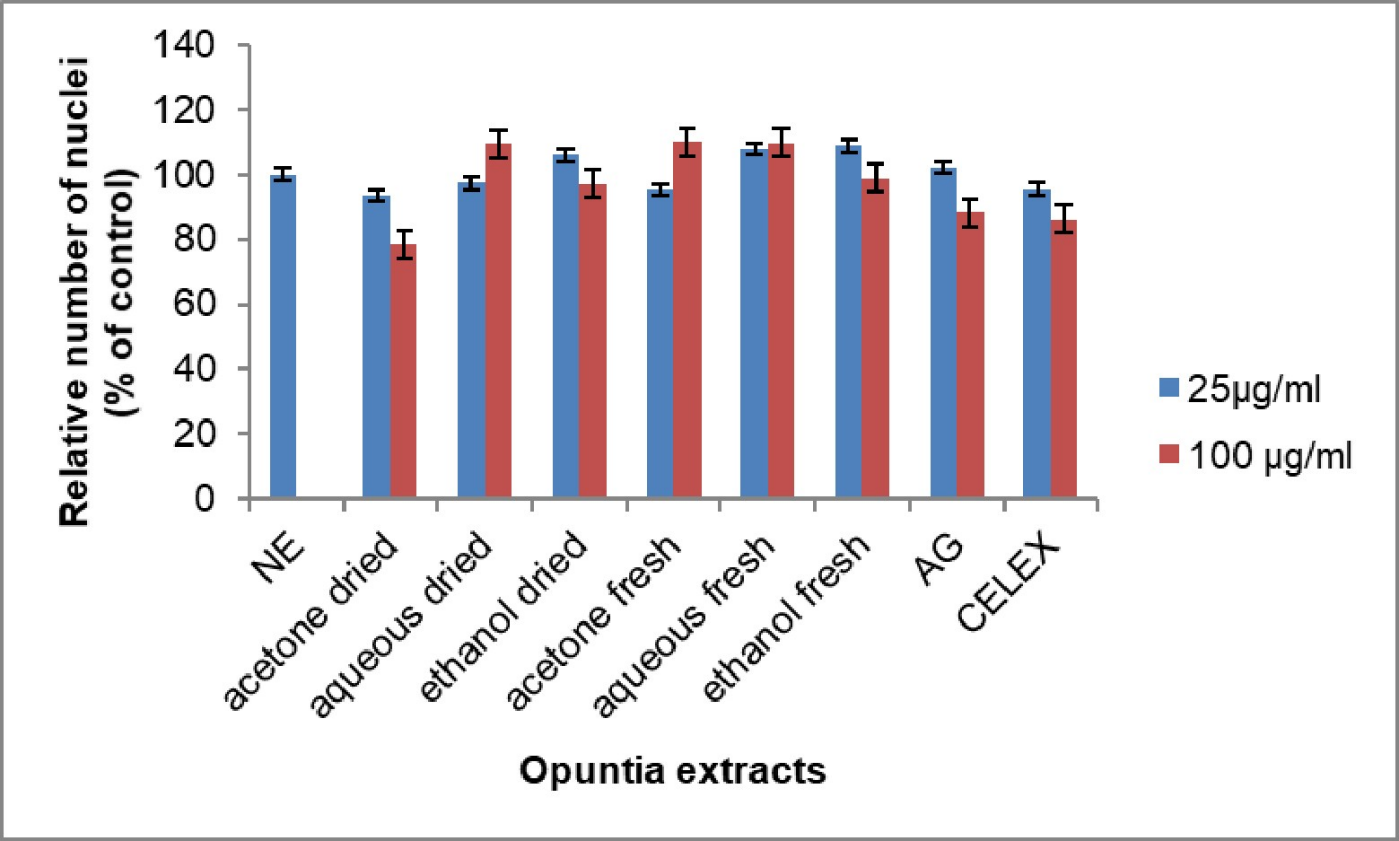
The effect of various *Opuntia stricta* extracts on RAW 264.7 cells density using the Hoechst stain. AG = Aminoguanidine. CELEX = Celecoxib. NE = No extract. Each value represents mean ± S.D (n = 3).

For the COX-2 assay, the numbers of cells that are viable were determined by the Hoechst staining of the nucleic acids as previously described. The Opuntia extracts had mild cytotoxic effects on the RAW 264.7 cells, and which occurred mainly at a concentration of 100 μM (Fig 8). The results also showed that the activity of Opuntia extracts was not as a result of a reduction in cell viability.

### Effect of *Opuntia stricta* extracts on LPS- induced nitrous oxide production

**Fig 9:**
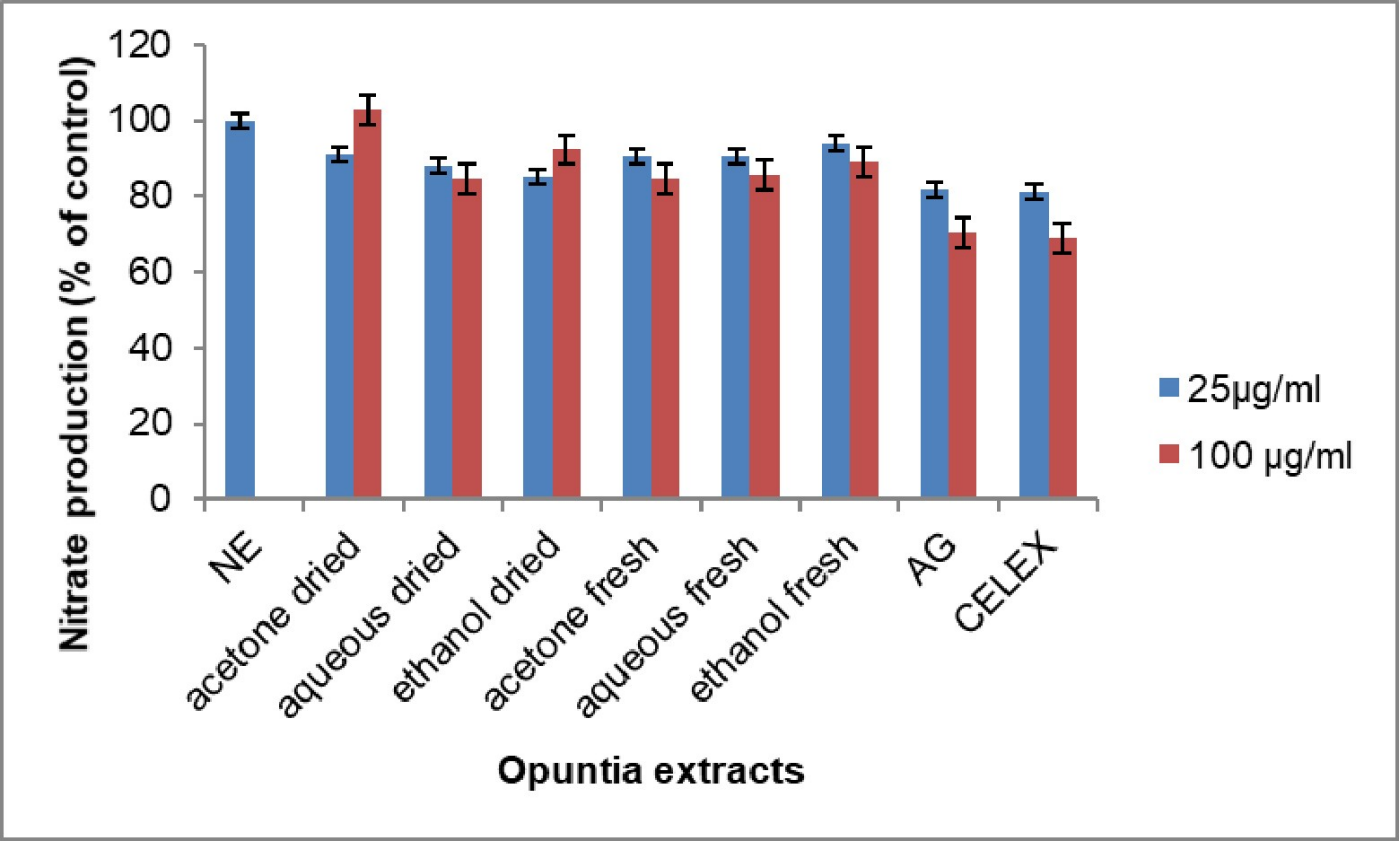
The effect of *Opuntia stricta* extracts on LPS- induced nitrous oxide production in RAW 264.7 cells. NE = No extract. AG = Aminoguanidine. CELEX = Celecoxib. NE = No extract. Values expressed as mean ± S.D (n = 3).

The various levels of nitrous oxide released were measured by an ELISA reader. When LPS alone was added to the RAW 264.7 cells, there was increased production of nitrous oxide as compared to the addition of the plant extracts and the controls. (Fig 9).

The level of nitrous oxide was significantly decreased in the plant extract groups and the controls when compared to LPS-induced only cells (p< 0.05). The level of nitrous oxide was however slightly higher in the plant extracts compared to the control group.

### Effect of *Opuntia stricta* extracts on COX-2 production

**Fig 10:**
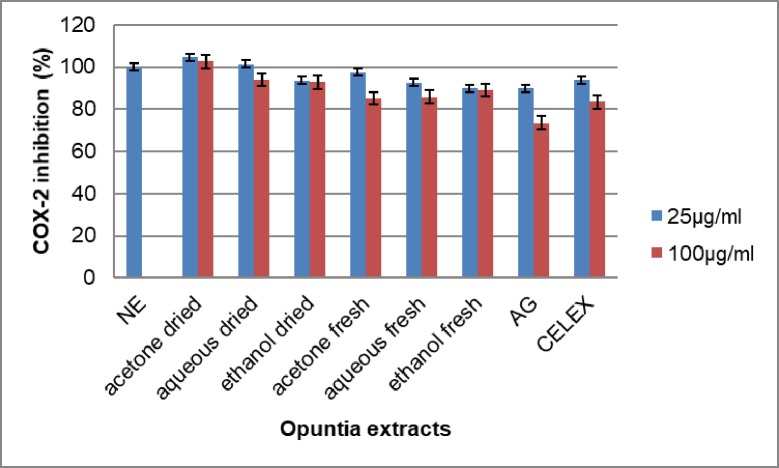
The effect of *Opuntia stricta* extracts on COX-2 expression in RAW 264.7 cells. NE = No extract. AG = Aminoguanidine. CELEX = Celecoxib. NE = No extract.Values expressed as mean ± S.D (n = 3).

The treatment of the RAW 264.7 cells with *Opuntia stricta* extracts caused a decrease in COX-2 expression in some of the wells (Fig 10). The acetone fresh and the aqueous fresh showed a COX-2 reduction of about 15% each which were comparable to celecoxib, a selective COX-2 inhibitor at 17%.

### Cytotoxic effect of *Opuntia stricta* extracts on U937 cell line

**Fig 11:**
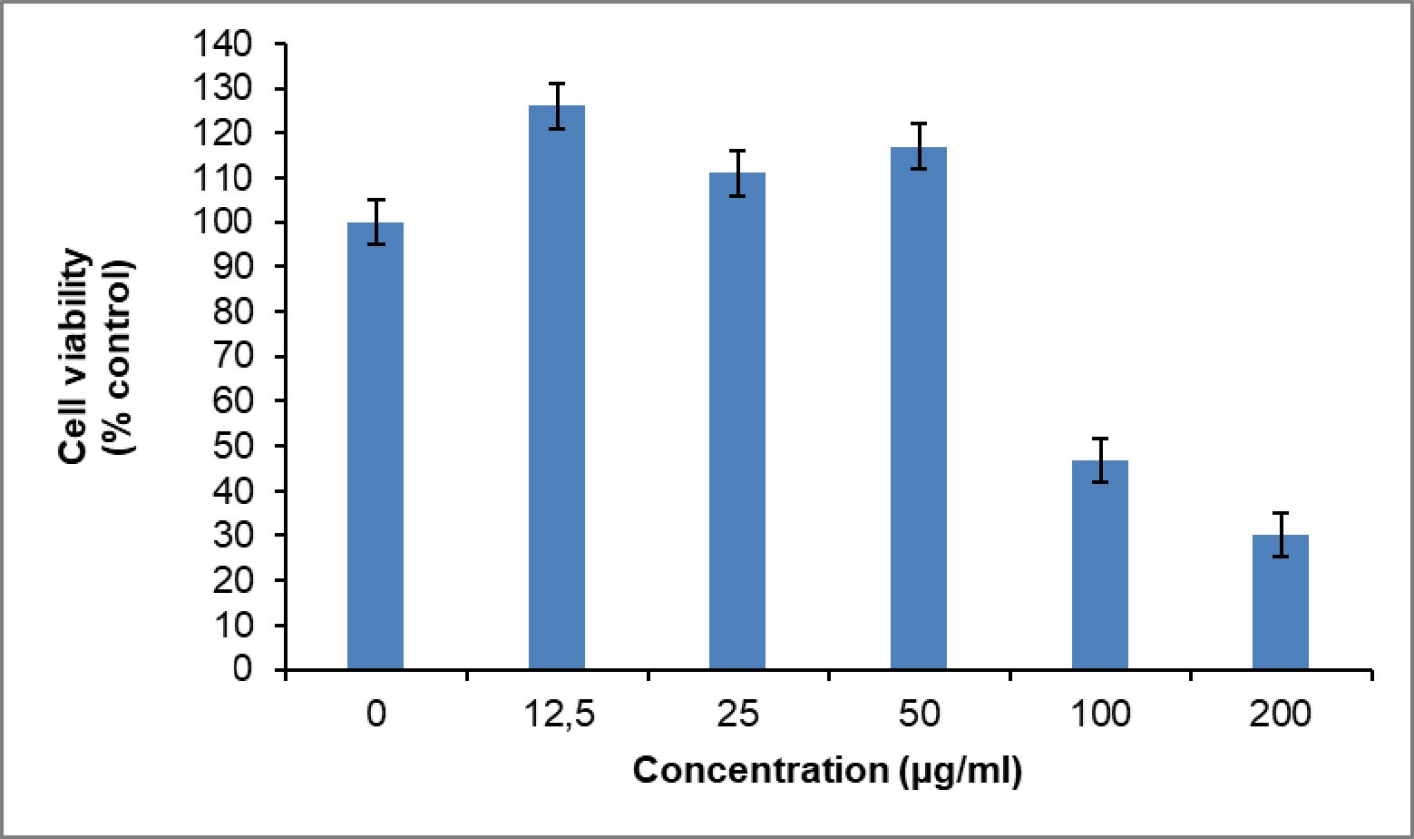
Cell viability of acetone fraction of the dried extract of *Opuntia stricta*. Values expressed as mean ± S.D (n = 3).

**Fig 12:**
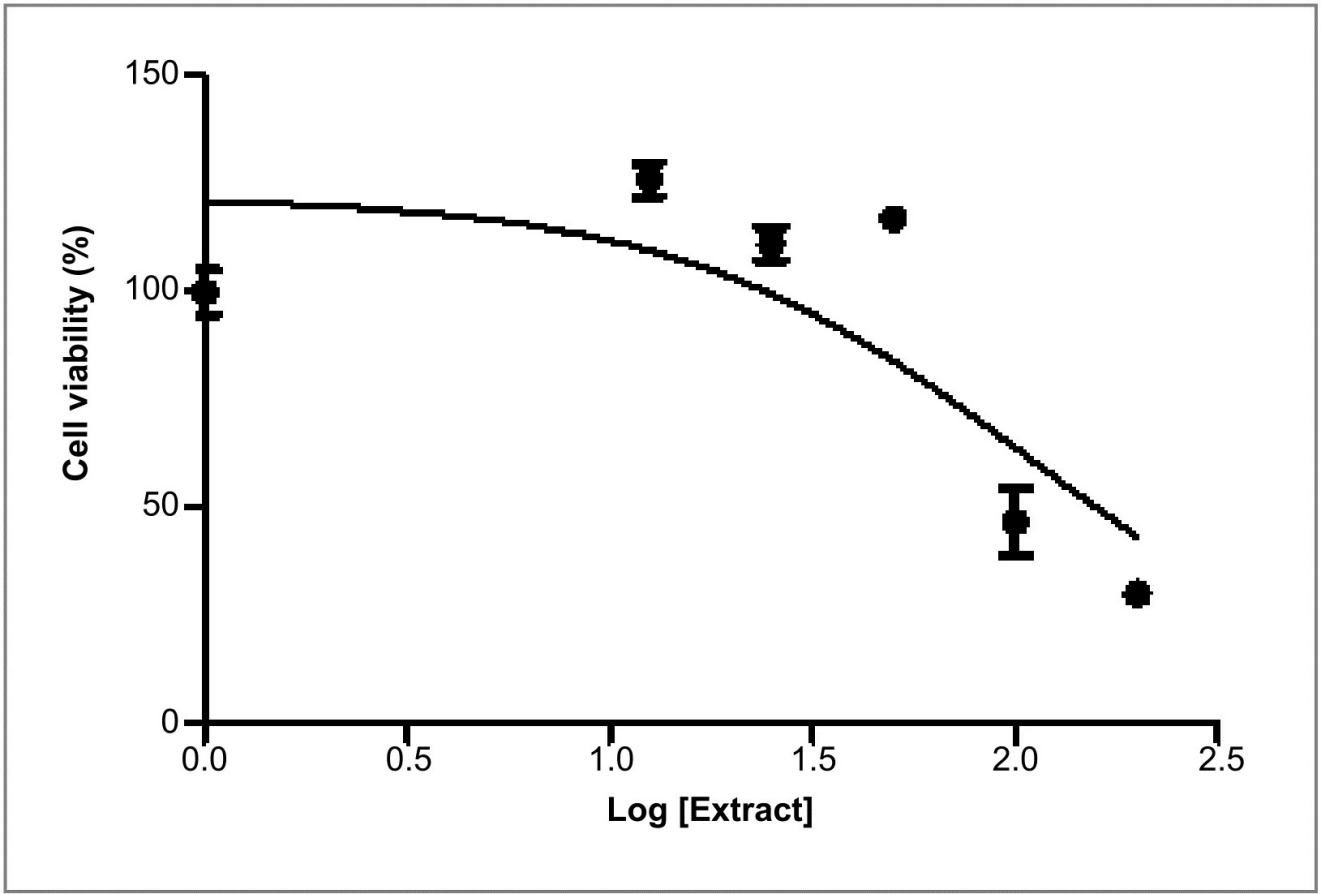
Log(inhibitor) vs response curve of acetone fraction of the dried extract of *Opuntia stricta* on U937 cell line.

The MTT assay carried out to determine the cytotoxic effects of the various *Opuntia stricta* extracts on the U937 cell line showed that after 48hrs none of the extracts displayed cytotoxic effect. However, at concentrations of 100 and 200μg/ml, the acetone dried extract displayed some activities as indicated in Table 4 and Fig 11. The IC50 was 110.1 μg/ml (Fig 12).

**Table 4:** Cytotoxic effect of Opuntia stricta cladode extracts on U937 cell line after 48hrs. Values expressed as mean ± S.D (n = 3).

Conc. (μg/ml)	% control	Acetone dried	Aqueous dried	Ethanol dried	Acetone fresh	Aqueous fresh	Ethanol fresh
0	**100**						
12.5		**126.06**± 5.5	**115.07**±9.2	**102.24**±19.6	**89.96**±8.8	**98.39**±20.4	**122.91**±6.9
25		**111.13**± 6.8	**112.69**±22.7	**99.86**±12.7	**105.1**±2.6	**95.98**±8.6	**109.25**±5
50		**116.96**± 2.6	**113.22**±12.5	**102.42**±17.8	**104.47**±4.9	**116.97**±16.4	**119.79**±13.3
100		**46.84**±13.6	**139.96**±23.8	**109.08**±12.3	**118.47**±4.6	**103.6**±4.5	**118.88**±2.6
200		**30.16**±0.8	**148.46**±27.7	**109.51**±9.15	**116.04**±4.9	**99.47**±4.5	**121.4**±10.7

## Discussion

The phytochemical composition of each extract differs significantly. The polyphenols (phenols, flavonoids, flavonol, proanthocyanidin, tannins) were the major compounds found in the various extracts of *Opuntia stricta*. They are also well reported to be strongly associated with antioxidant capacity[64]. Phenolic compounds are known to scavenge free radicals, neutralizing their ability to cause cellular damage[65]. These ability to prevent cellular oxidative stress is suggested as a means to prevent chronic diseases[66]. Flavonoids have been studied extensively, and are known to have antioxidative, anti-inflammatory and antineoplastic effects [67]. The cladodes of some cactus plants have been shown to produce some high amounts of flavonoids and flavonoid-like compounds, including isoquercetin and nicotiflorin[68]. Nicotiflorin has been shown to have anti-inflammatory and neuroprotective properties against cerebral ischaemia [69],while isoquercetin is currently under clinical investigation as an antithrombotic in certain cancer patients (NCT02195232).The mode of action of polyphenols is through the activation of several signalling pathways including NF-κB, and MAPK p38/JNK which exerts antioxidative and anti-inflammatory effects [70].

Saponin, another type of phytochemicals, are glycosides that contain triterpenoid or spirostane aglycones. There are two main types of saponin, triterpenoid saponins and steroidal saponins. Saponins have been reported to exert different pharmacological actions such as anti-inflammatory[71], immunoregulatory [72] and also antineoplastic [73]. They are also known to display antioxidant properties[74].The saponin content from the pulverized sample of *Opuntia stricta* was very high compared to the control, which can easily be explained since carbohydrates are the second highest constituents found in the cladodes of Opuntia after water[23]. Since antioxidants play a role in the management of some free radical-related ailments like cancers, the cladodes of *Opuntia stricta* with its rich level of phytochemicals can be used as either dietary or complementary agents.

### Vitamins

Vitamins A, E and C were present in the cladodes of *Opuntia stricta*. Vitamins are known to play a role in cellular homeostasis and well-being. They do this by neutralizing ROS development[75] and as such can play a role as antioxidant adjuvants. Vitamins have been used in cancer treatment to mop up free radicals generated during chemotherapy and radiotherapy[76]. The use of high dose retinol supplementation during chemotherapy reduces adverse effects of intestinal malabsorption in children with leukaemia and lymphoma[77]. The Ten-Eleven Translocation-2 (TET2) enzyme activity is known to be enhanced in the presence of vitamin C (TET2 mutation is common in AML), and as such vitamin C has been reported to induce differentiation in leukaemia stem cells [78]. vitamin E is an important antioxidant that has been reported to reduce oxidative stress and lipid peroxidation[79]. These vitamins are present in the cladodes of *Opuntia stricta*, and so makes the plant an attractive choice for antioxidant therapy.

### Anti-inflammatory

*Opuntia stricta* extracts caused a decrease in the expression of nitric oxide by lipopolysaccharide-treated RAW 264.7 macrophages cell and COX-2 expression. This proves the anti-inflammatory activity of *Opuntia stricta* extracts. However, while some extracts had an anti-inflammatory effect, a few others caused a slight increased production of nitric oxide and COX-2. The slight increase was more evident in the dry acetone extract (Figs 9 and 10). The anti-inflammatory activity of *Opuntia spp*. is well reported [80][81]. Opuntia spp. are also known to induce pro-inflammatory cytokines. Extracts of *Opuntia polyacantha* has been shown to induce production of ROS, nitric oxide and interleukin 6 [82]. The induction of nitric oxide, COX-2 and some inflammatory cytokines are known to play a role in oxidative stress-induced inflammation[83]. Some cancers are known to overexpression COX-2[84][85]. COX-2 is thus known to mediate cell metastasis [86] as well as mediate immune tolerance through the Indoleamine 2, 3-dioxygenase 1 (IDO1) pathway[87]. This expression of COX-2 is reported to be induced by ROS through activation of NF-κB and ERK1/2 in macrophages[88]. Thus selective COX-2 inhibitors can have a role to play in cancer management [89].

Some of the compounds found in the essential oil also have good antioxidant and anti-inflammatory properties (Table 3). Compounds like germacrene-D, globulol and caryophyllene also found in *Opuntia stricta* have been shown to have good anti-inflammatory properties[90]. This explains the anti-inflammatory effect shown by *Opuntia stricta*.

### Cytotoxicity

While the essential oil of *Opuntia stricta* contains some cytotoxic compounds, the non-cytotoxic effect of the cladodes of *Opuntia stricta* against U937 cell lines may just be that the compounds were not in sufficient amounts. This can also be explained by the low levels of phytochemicals it yielded in this study. This non-cytotoxicity was also observed by Gebresamuel and Gebre-Mariam [91] in their study of *Opuntia stricta* and *Opuntia ficus indica* cladodes. Recently Harrabi *et al*. reported the cytoprotective effect of *Opuntia stricta* cladodes extracts on HepG2 cells[92]. Apart from the level of the phytochemicals, seasonal variation, soil type, and other variables can also affect the level of these compounds in the plant [93]; while the literature quoted higher yields during the summer, the *Opuntia stricta* cladodes in this study were harvested in the winter. In a study done by Alves *et al*. on different *Opuntia spp*. including *Opuntia stricta*, seasonal variations affected the distribution of phytochemicals and nutrients in the plant, and none of the cladodes displayed cytotoxicity against the cell lines used[94].

However, the ethyl acetate fraction of *Opuntia stricta* flower extract was reported to have antineoplastic activity against HepG2 cell line [95]. Betalains, which are water-soluble nitrogenous pigments present in flowers and fruits of *Opuntia spp*., have also been reported to have good antineoplastic activity against some cancers[96][97]. Betalains from the fruit of *Opuntia ficus indica* was shown to induce apoptosis in chronic myeloid leukaemia, K562 cell line [98]*Opuntia stricta* is known to have the highest total betalain content among all the *Opuntia spp*. studied[23]. So it is possible some other parts of the plants have compounds with antineoplastic activity.

## Conclusion

The results show that the cladodes of *Opuntia stricta* are a good source of vitamins, phytochemicals and essential oil with some medicinal benefits. This study reveals that *Opuntia stricta* have antioxidative and anti-inflammatory properties. It is also mildly cytotoxic, which makes it safe to consume as food. These properties make *Opuntia stricta* a good choice as a complementary source to use against diseases that involve oxidative stress. This study is expected to spur more research into *Opuntia stricta* for its therapeutic and palliative uses.

## Supporting information

S1 File UFH Final data Dec 2017 (1). This file contains all the raw data from which the graphs were drawn.
(XLSX)

## Abbreviations

DPPH: Alpha, alpha-diphenyl- β-picrylhydrazyl
H_2_O_2_: Hydrogen peroxide
NO: Nitric oxide
COX-2: Cyclo-oxygenase 2
FBS: Fetal bovine serum
BSA: Bovine serum albumin
DMEM: Dulbecco’s Modified Eagle Medium
MTT: 3-(4,5dimethylthiazol-2-yl)-2,5diphenyltetrazoliumbromide
DMSO: Dimethyl sulfoxide
LPS: Lipopolysarccharide
ANOVA: Analysis of variance
CELEX: Celecoxib
AG: Aminoguanidine
ROS: Reactive oxygen species
OS: Oxidative stress

## References

[1] Valko M, Leibfritz D, Moncol J, Cronin MT, Mazur M, Telser J: Free radicals and antioxidants in normal physiological functions and human disease. Int J Biochem Cell Biol. 2007; 39:44–84. 10.1016/j.biocel.2006.07.001 16978905

[2] Noori S. An Overview of Oxidative Stress and Antioxidant Defensive System. 2012; 1:413 10.4172/scientificreports.413

[3] Schraufstatter I, Hyslop PA, Jackson JH, Cochrane CG. Oxidant-induced DNA damage of target cells. J Clin Invest 1988;82:1040–1050. 10.1172/JCI113660 2843565 PMC303618

[4] Singh U and Jialal L. Oxidative stress and atherosclerosis. Pathophysiology. 2006; 13(3):129–42. 10.1016/j.pathophys.2006.05.002 16757157

[5] Csányi G, Miller FJ. Oxidative Stress in Cardiovascular Disease. International Journal of Molecular Sciences. 2014;15(4):6002–6008. 10.3390/ijms15046002 24722571 PMC4013610

[6] Giacco F, Brownlee M. Oxidative stress and diabetic complications. Circulation research. 2010;107(9):1058–1070. 10.1161/CIRCRESAHA.110.223545 21030723 PMC2996922

[7] Salzanoa S, Checconia P, Hanschmannc EM, Lilligc CH, Bowlerd LD, Chane P, et al. Linkage of inflammation and oxidative stress via release of glutathionylated peroxiredoxin-2, which acts as a danger signal. PNAS. 2014; 111(33)12157–12162. 10.1073/pnas.1401712111 25097261 PMC4143057

[8] Gupta RK, Patel AK, Shah N, Choudhary AK, Jha UK, Yadav UC, et al Oxidative Stress and Antioxidants in Disease and Cancer: A Review. Asian Pac J Cancer Prev. 2014; 15 (11), 4405–4409. 24969860 10.7314/apjcp.2014.15.11.4405

[9] Trachootham D, Lu W, Ogasawara MA, Valle NR-D, Huang P. Redox Regulation of Cell Survival. Antioxidants & Redox Signaling. 2008;10(8):1343–1374. 10.1089/ars.2007.1957 18522489 PMC2932530

[10] Trachootham D, Alexandre J, Huang P: Targeting cancer cells by ROS-mediated mechanisms: a radical therapeutic approach? Nat Rev Drug Discov 2009; 8:579–591. 10.1038/nrd2803 19478820

[11] Onodera Y, Teramura T, Takehara T, Shigi K, Fukuda K. Reactive oxygen species induce Cox-2 expression via TAK1 activation in synovial fibroblast cells. FEBS Open Bio. 2015;5:492–501. 10.1016/j.fob.2015.06.001 26110105 PMC4476901

[12] Sobolewski C, Cerella C, Dicato M, Ghibelli L and Marc Diederich. “The Role of Cyclooxygenase-2 in Cell Proliferation and Cell Death in Human Malignancies,” International Journal of Cell Biology. 2010 10.1155/2010/215158 20339581 PMC2841246

[13] Iachininoto MG (2013). Cyclooxygenase-2 (COX-2) Inhibition Constrains Indoleamine 2,3-Dioxygenase 1 (IDO1) Activity in Acute Myeloid Leukaemia Cells. Molecules, 18, 10132–10145. 10.3390/molecules180910132 23973990 PMC6270179

[14] Arts IC, Hollman PC. Polyphenols and disease risk in epidemiologic studies. Am J Clin Nutr. 2005; 81: 317–25. 10.1093/ajcn/81.1.317S 15640497

[15] Kim YS, Young MR, Bobe G, Colburn NH, and Milner JA. Bioactive food components, inflammatory targets, and cancer prevention. Cancer Prevention Research. 2009; 2 (3): 200–208. 10.1158/1940-6207.CAPR-08-0141 19258539 PMC3449301

[16] Reuter Simone, Gupta Subash C., Chaturvedi Madan M., and Aggarwal Bharat B. Oxidative stress, inflammation, and cancer: How are they linked? Free Radic Biol Med. 2011; 49(11): 1603–1616. 10.1016/j.freeradbiomed.2010.09.006 PMC2990475 20840865

[17] Mishra A, Sharma AK, Kumar S, Saxena AK, and Pandey AK. Bauhinia variegata Leaf Extracts Exhibit Considerable Antibacterial, Antioxidant, and Anticancer Activities. BioMed Research International. 2013, 10 10.1155/2013/915436 24093108 PMC3777169

[18] Schuurman AG, Goldbohm RA, Brants HA, van den Brandt PA: A prospective cohort study on intake of retinol, vitamins C and E, and carotenoids and prostate cancer risk (Netherlands). Cancer Causes Control. 2002; 13:573–582. 12195647 10.1023/a:1016332208339

[19] Cimino L, Dolgalev I, Wang Y, Yoshimi A, Martin GH, Wang J, et al Restoration of TET2 Function Block Aberrant Self-Renewal and Leukemia Progression. Cell. 2017; 170(6): 1079–1095. 10.1016/j.cell.2017.07.032 28823558 PMC5755977

[20] Montecinos V, Guzmán P, Barra V, Villagrán M, Muñoz-Montesino C, Sotomayor K, et al Vitamin C is an Essential Antioxidant That Enhances Survival of Oxidatively Stressed Human Vascular Endothelial Cells in the Presence of a Vast Molar Excess of Glutathione. The Journal of Biological Chemistry. 2007; 282:21 15506–15515. 10.1074/jbc.M608361200 17403685

[21] Olajuyigbe OO and Afolayan AJ. Phenolic content and antioxidant property of the bark extracts of Ziziphus mucronata Willd. subsp. mucronata Willd. BMC Complementary and Alternative Medicine. 2011; 11:130 10.1186/1472-6882-11-130 22176659 PMC3295714

[22] https://www.cabi.org/isc/datasheet/120172. Accessed 19th April 2017.

[23] del Socorro Santos Díaz M, Barba de la Rosa AP, Héliès-Toussaint C, Guéraud F and Nègre-Salvayre A. Opuntia spp.: Characterization and Benefits in Chronic Diseases. Oxidative Medicine and Cellular Longevity. 2017; 10.1155/2017/8634249. PMC5401751 28491239

[24] Osuna-Martínez U, Reyes-Esparza J, Rodríguez-Fragoso L. Cactus (Opuntia ficus-indica): A Review on its Antioxidants Properties and Potential Pharmacological Use in Chronic Diseases. Nat Prod Chem Res. 2014; 2: 153.

[25] Osorio-Esquivel O, Ortiz-Moreno A, Garduño-Siciliano L, Alvarez VB, Hernández-Navarro MD. Antihyperlipidemic effect of methanolic extract from Opuntia joconostle seeds in mice fed a hypercholesterolemic diet. Plant Foods Hum Nutr. 2012; 67: 365–370. 10.1007/s11130-012-0320-2 23135897

[26] Kim J, Soh SY, Shin J, Cho CW, Choi YH, Nam SY. Bioactives in cactus (Opuntia ficus-indica) stems possess potent antioxidant and pro-apoptotic activities through COX-2 involvement. J Sci Food Agric. 2015; 95(13):2601–6. 10.1002/jsfa.6968 25345579

[27] Hahm S-W, Park J, Son Y-S. Opuntia humifusa partitioned extracts inhibit the growth of U87MG human glioblastoma cells. Plant Foods for Human Nutrition. 2010;65(3):247–252. 10.1007/s11130-010-0188-y 20814744

[28] Vijay T and Bhambar RS. Estimation of Total Phenol, Tannin, Alkaloid and Flavonoid in Hibiscus Tiliaceus Linn. Wood Extracts. Research and Reviews: Journal of Pharmacognosy and Phytochemistry. 2014; 2 (4): 41–47.

[29] Wintola O.A and Afolayan A.J. Phytochemical constituents and antioxidant Activities of the whole leaf extract of Aloe ferox Mill. Pharmacognosy Magazine. 2011; 7: 325–33. 10.4103/0973-1296.90414 22262936 PMC3261067

[30] Cáceres-Mella A., Peña-Neira Á., Narváez-Bastias J., Jara-Campos C., López-Solís R. and Canals J. M. Comparison of analytical methods for measuring proanthocyanidins in wines and their relationship with perceived astringency. International Journal of Food Science & Technology. 2013; 48: 2588–2594.

[31] Unuofin JO, Otunola GA, Afolayan JA. Phytochemical screening and in vitro evaluation of antioxidant and antimicrobial activities of Kedrostis africana (L.) Cogn. Asian Pac J Trop Biomed. 2017; 7(10): 901–908.

[32] Omoruyi BE, Bradley G and Afolayan AJ. Antioxidant and phytochemical properties of Carpobrotus edulis (L.) bolus leaf used for the management of common infection in HIV/AIDS patients in Eastern Cape Province. BMC Complementary and Alternative Medicine. 2012; 12:215 10.1186/1472-6882-12-215 23140206 PMC3528461

[33] Olajuyigbe OO and Afolayan AJ. Phenolic content and antioxidant property of the bark extracts of Ziziphus mucronata Willd. subsp. mucronata Willd. BMC Complementary and Alternative Medicine. 2011, 11:130 10.1186/1472-6882-11-130 22176659 PMC3295714

[34] Boora F, Chirisa E, and Mukanganyama S. Evaluation of Nitrite Radical Scavenging Properties of Selected Zimbabwean Plant Extracts and Their Phytoconstituents. Journal of Food Processing. 2014; dx.doi.org/10.1155/2014/918018.

[35] Oyedemi SO, Bradley G and Afolayan AJ. In vitro and In vivo antioxidant activities of aqueous extract of Strychonos henningsii Gilg. Afri J Pharm Pharmacol. 2010; 4:70–8.

[36] Ahmed D, Khan MM and Saeed R. Comparative Analysis of Phenolics, Flavonoids, and Antioxidant and Antibacterial Potential of Methanolic, Hexanic and Aqueous Extracts from Adiantum caudatum Leaves. Antioxidants. 2015; 4: 394–409. 10.3390/antiox4020394 26783712 PMC4665467

[37] Onyesife CO, Ogugua VN and Anaduaka EG. Investigation of some important phytochemicals, vitamins and mineral constituents of ethanol leaves extract of Piper Nigrum. Annals of Biological Research. 2014; 5 (6):20–25.

[38] Igwe OU and Okwu DE. Investigation of the chemical composition of brachystegia eurycoma harms plant parts used in herbal medicine. Int. Res J Pharm. App Sci. 2013; 3(6):51–55.

[39] Asokkumar S, Naveenkumar C, Raghunandhakumar S, Kamaraj S, Anandakumar P, Jagan S, Devaki T. Antiproliferative and antioxidant potential of beta-ionone against benzo(a)pyrene-induced lung carcinogenesis in Swiss albino mice. Mol Cell Biochem. 2012;363(1–2):335–45. 10.1007/s11010-011-1186-6 22187222

[40] Aydin E., Türkez H. & Taşdemir Ş. Anticancer and Antioxidant Properties of Terpinolene in Rat Brain Cells. Archives of Industrial Hygiene and Toxicology. 2013; 64(3): 415–424. 10.2478/10004-1254-64-2013-2365 24084350

[41] Aydin E, Turkez H and Geyikoglu F. Antioxidative, anticancer and genotoxic properties of α-pinene on N2a neuroblastoma cells. Biologia. 2013; 68 (5): 1004–1009.

[42] Kim DS, Lee HJ, Jeon YD, Han HY, Kee JY, Kim HJ et al Alpha-Pinene Exhibits Anti-Inflammatory Activity Through the Suppression of MAPKs and the NF-κB Pathway in Mouse Peritoneal Macrophages. American Journal of Chinese Medicine. 2015;43(4):731–42. 26119957 10.1142/S0192415X15500457

[43] Bendaoud H, Romdhane M, Souchard JP, Cazaux S, Bouajila A. Chemical Composition and Anticancer and Antioxidant Activities of *Schinus Molle* L. and *Schin*us *Terebinthifolius* Raddi Berries Essential Oils. Journal of Food Science. 2010; 75(6): 466–472. 10.1111/j.1750-3841.2010.01711.x 20722898

[44] Kim MJ, Yang KW, Kim SS, Park SM, Park KJ, Kim KS et al Chemical composition and anti-inflammation activity of essential oils from Citrus unshiu flower. Natural Product Communications. 2014;9(5):727–30. 25026734

[45] Iwasaki K, Zheng Y-W, Murata S, Ito H, Nakayama K, Kurokawa T, et al Anticancer effect of linalool *via* cancer-specific hydroxyl radical generation in human colon cancer. World Journal of Gastroenterology. 2016;22(44):9765–9774. 10.3748/wjg.v22.i44.9765 27956800 PMC5124981

[46] Peana AT, Marzocco S, Popolo A, Pinto A. (-)-Linalool inhibits in vitro NO formation: Probable involvement in the antinociceptive activity of this monoterpene compound. Life Sciences. 2006; 11;78(7):719–23. 10.1016/j.lfs.2005.05.065 16137709

[47] Aydin E, Turkez H, Tasdemir S. Anticancer and antioxidant properties of terpinolene in rat brain cells. Arh Hig Rada Toksikol. 2013;64(3):415–24. 10.2478/10004-1254-64-2013-2365 24084350

[48] Macedo EMA, Santos WC, Sousa BP, Lopes EM, Piauilino CA, Cunha FV et al. Association of terpinolene and diclofenac presents antinociceptive and anti-inflammatory synergistic effects in a model of chronic inflammation. Brazilian Journal of Medical and Biological Research. 2016;49(7):e5103. 10.1590/1414-431X20165103 PMC4918787 27332775

[49] Ansari M, Emami S. β-Ionone and its analogs as promising anticancer agents. European Journal of Medicinal Chemistry. 2016; 10;123:141–154. 10.1016/j.ejmech.2016.07.037 27474930

[50] Kang CH, Jayasooriya RG, Choi YH, Moon SK, Kim WJ, Kim GY. β-Ionone attenuates LPS-induced pro-inflammatory mediators such as NO, PGE2 and TNF-α in BV2 microglial cells via suppression of the NF-κB and MAPK pathway. Toxicology In Vitro. 2013;27(2):782–7. 10.1016/j.tiv.2012.12.012 23268108

[51] Albano SM, Lima AS, Miguel GM, Pedro LG, Barosso GJ, Figueiredo CA. Antioxidant, Anti-5-lipoxygenase and Antiacetylcholinesterase Activities of Essential Oils and Decoction Waters of Some Aromatic Plants. Rec. Nat. Prod. 2012; 6(1): 35–48.

[52] Türkez H, Çelik K, Toğar B. Effects of copaene, a tricyclic sesquiterpene, on human lymphocytes cells in vitro. Cytotechnology. 2014;66(4):597–603. 10.1007/s10616-013-9611-1 24287609 PMC4082788

[53] Turkez H, Togar B, Tatar A, Geyıkoglu F, Hacımuftuoglu A. Cytotoxic and cytogenetic effects of *α*-copaene on rat neuron and N2a neuroblastoma cell lines. Biologia. 2014;69 (7): 936–942.

[54] Basholli-Salihu M, Schuster R, Hajdari A, Mulla D, Viernstein H, Mustafa B et al Phytochemical composition, antiinflammatory activity and cytotoxic effects of essential oils from three Pinus spp. Pharmaceutical Biology. 2017; 55(1): 1553–1560. 10.1080/13880209.2017.1309555 28385055 PMC6130611

[55] Zhang Y, Wang X, Ma L, Dong L, Zhang X, Chen J et al Anti-inflammatory, antinociceptive activity of an essential oil recipe consisting of the supercritical fluid CO2 extract of white pepper, long pepper, cinnamon, saffron and myrrh in vivo. Journal *of oleo science*. 2014;63(12):1251–60. 25263165 10.5650/jos.ess14061

[56] Correa MG, Couto JS, and Teodoro AJ. Anticancer Properties of Psidium guajava—a Mini-Review. Asian Pac J Cancer Prev. 2016; 17 (9): 4199–4204. 27797217

[57] Pinheiro BG, Silva AS, Souza GE, Figueiredo JG, Cunha FQ, Lahlou S et al Chemical composition, antinociceptive and anti-inflammatory effects in rodents of the essential oil of Peperomia serpens (Sw.) Loud. J Ethnopharmacol. 2011 11 18;138(2):479–86. 10.1016/j.jep.2011.09.037 21971207

[58] Salleh WM, Ahmad F. Antioxidant and Anti-inflammatory Activities of Essential Oils of Actinodaphne macrophylla and A. pruinosa (Lauraceae). Natural productions communications. 2016;11(6):853–5. 27534134

[59] Boligon AA, Schwanz TG, Piana M, Bandeira RV, Frohlich JK, de Brum TF et al Chemical composition and antioxidant activity of the essential oil of Tabernaemontana catharinensis A. DC. leaves, Natural Product Research. 2013; 27(1): 68–71 10.1080/14786419.2011.653971 22273350

[60] Kim DH, Park MH, Choi YJ, Chung KW, Park CH, Jang EJ, et al. Molecular Study of Dietary Heptadecane for the Anti-Inflammatory Modulation of NF-kB in the Aged Kidney. PLoS ONE. 2013;8(3):e59316 10.1371/journal.pone.0059316 23555655 PMC3608635

[61] Kazemi M. Phenolic profile, antioxidant capacity and anti-inflammatory activity of Anethum graveolens L. essential oil. Nat Prod Res. 2015;29(6):551–3. 10.1080/14786419.2014.951934 25154406

[62] Boddu SH, Alsaab H, Umar S, Bonam SP, Gupta H, and Ahmed S. Anti-inflammatory effects of a novel ricinoleic acid poloxamer gel system for transdermal delivery. Int J Pharm. 2015;479(1):207–11 10.1016/j.ijpharm.2014.12.051 25542985

[63] Hansen JF, Bendtzen K, Boas M, Frederiksen H, Nielsen CH, Rasmussen ÅK, et al Influence of Phthalates on Cytokine Production in Monocytes and Macrophages: A Systematic Review of Experimental Trials. PLoS ONE. 2015; 10(3): e0120083 10.1371/journal.pone.0120083 25811352 PMC4374770

[64] Williams RJ, Spencer JP, Rice-Evans C: Flavonoids: Antioxidants or signaling molecules? Free Rad Biol Med 2004;36:838–849. 10.1016/j.freeradbiomed.2004.01.001 15019969

[65] Dib H, Beghdad MC, Belarbi M, Seladji M & Ghalem M. Antioxidant activity of phenolic compounds of the cladodes of Opuntia ficus-indica mill. from northwest Algeria. International Journal of Medicine and Pharmaceutical Sciences. 2013; 3: 147–158.

[66] Reinisalo M, Kårlund A, Koskela A, Kaarniranta K, and Karjalainen RO.Polyphenol Stilbenes: Molecular Mechanisms of Defence against Oxidative Stress and Aging-Related Diseases. Oxidative Medicine and Cellular Longevity.2015; 24 10.1155/2015/340520 PMC4477219 26180583

[67] Aggarwal BB, Harikumar KB. Potential Therapeutic Effects of Curcumin, the Anti-inflammatory Agent, Against Neurodegenerative, Cardiovascular, Pulmonary, Metabolic, Autoimmune and Neoplastic Diseases. The international journal of biochemistry & cell biology. 2009; 41(1):40–59. 18662800 10.1016/j.biocel.2008.06.010 PMC2637808

[68] El-Mostafa K, El Kharrassi Y, Badreddine A, Andreoletti P, Vamecq J, El Kebbaj MS, et al Nopal Cactus (Opuntia ficus-indica) as a Source of Bioactive Compounds for Nutrition, Health and Disease. Molecules. 2014; 19, 14879–14901 10.3390/molecules190914879 25232708 PMC6270776

[69] Li R.; Guo M.; Zhang G.; Xu X.; Li Q. Nicotiflorin reduces cerebral ischemic damage and upregulates endothelial nitric oxide synthase in primarily cultured rat cerebral blood vessel endothelial cells. Journal of Ethnopharmacology. 2006;107, 143–150. 10.1016/j.jep.2006.04.024 16806761

[70] Varela-López A, Bullón P, Giampieri F and Quiles JL. Non-Nutrient, Naturally Occurring Phenolic Compounds with Antioxidant Activity for the Prevention and Treatment of Periodontal Diseases. Antioxidants. 2015; 4(3), 447–481. 10.3390/antiox4030447 26783837 PMC4665427

[71] Xiang L, Yi X, Wang Y, He X. Antiproliferative and anti-inflammatory polyhydroxylated spirostanol saponins from Tupistra chinensis. Sci. Rep. 2016, 6. 10.1038/srep31633 PMC4987684 27530890

[72] Khan M., Maryam A., Zhang H., Mehmood T., Ma T. Killing cancer with platycodin D through multiple mechanisms. J. Cell. Mol. Med. 2016, 20, 389–402. 10.1111/jcmm.12749 26648178 PMC4759477

[73] Chen MF, Huang SJ, Huang CC, Liu PS, Lin KI, Liu CW, et al Saikosaponin D induces cell death through caspase-3-dependent, caspase-3-independent and mitochondrial pathways in mammalian hepatic stellate cells. BMC *Cancer* 2016, 16. 10.1186/s12885-016-2599-0 PMC4962421 27461108

[74] Ashraf MF, Abd Aziz M, Stanslas J, Ismail I, and Abdul Kadir M. Assessment of Antioxidant and Cytotoxicity Activities of Saponin and Crude Extracts of Chlorophytum borivilianum. The Scientific World Journal. 2013;7 10.1155/2013/216894 24223502 PMC3809374

[75] Fuchs-Tarlovsky V. Role of antioxidants in cancer therapy. Nutrition. 2013;29(1):15–21. 10.1016/j.nut.2012.02.014 22784609

[76] Yasueda A, Urushima H and Ito T. Efficacy and Interaction of Antioxidant Supplements as Adjuvant Therapy in Cancer Treatment. Integr Cancer Ther. 2016; 15(1): 17–39. 10.1177/1534735415610427 26503419 PMC5736082

[77] Dagdemir A, Yildirim H, Aliyazicioglu Y, Kanber Y, Albayrak D, Acar S. Does vitamin A prevent high-dose-methotrexate-induced D-xylose malabsorption in children with cancer? Support Care Cancer. 2004;12:263–267. 10.1007/s00520-004-0591-8 14767751

[78] Miller PG and Ebert BL. Vitamin C regulates stem cells and cancer. Nature. 2017; 549: 462–463. 10.1038/nature23548 28869971

[79] Khor SC, Abdul Karim N, Wan Ngah WZ, Mohd Yusof YA, Makpol S. Vitamin E in Sarcopenia: Current Evidences on Its Role in Prevention and Treatment. Oxidative Medicine and Cellular Longevity. 2014;2014:914853 10.1155/2014/914853 25097722 PMC4109111

[80] Siddiqui F. Opuntia dillenii cladode: Opuntiol and Opuntioside attenuated cytokines and eicosanoids mediated inflammation. Journal of Ethnopharmacology.2016;182:221–234. 10.1016/j.jep.2016.02.016 26900126

[81] Garoby-Salom S, Guéraud F, Camaré C, de la Rosa AP, Rossignol M, Santos Díaz Mdel S, et al Dietary cladode powder from wild type and domesticated Opuntia species reduces atherogenesis in apoE knock-out mice. Journal of Physiology and Biochemistry. 2016;72(1):59–70. 10.1007/s13105-015-0461-5 26704378

[82] Schepetkin IA, Xie G, Kirpotina LN, Klein RA, Jutila MA, Quinn MT. Macrophage immunomodulatory activity of polysaccharides isolated from Opuntia polyacantha. Int Immunopharmacol 2008; 1455–66. 10.1016/j.intimp.2008.06.003 18597716 PMC2614931

[83] Hussain SP, Harris CC. Inflammation and cancer: an ancient link with novel potentials. Int J Cancer 2007;121:2373–2380. 10.1002/ijc.23173 17893866

[84] Bernard MP, Bancos S, Sime PJ, Phipps RP. Targeting Cyclooxygenase-2 in Hematological Malignancies: Rationale and Promise. Current pharmaceutical design. 2008;14(21):2051–2060. 18691115 10.2174/138161208785294654 PMC2745246

[85] Jana D, Sarkar DK, Ganguly S, Saha S, Sa G, Manna AK, et al Role of Cyclooxygenase 2 (COX-2) in Prognosis of Breast Cancer. Indian Journal of Surgical Oncology. 2014;5(1):59–65. 10.1007/s13193-014-0290-y 24669166 PMC3964242

[86] Sobolewski C, Cerella C, Dicato M, Ghibelli L and Marc Diederich. “The Role of Cyclooxygenase-2 in Cell Proliferation and Cell Death in Human Malignancies,” International Journal of Cell Biology. 2010 10.1155/2010/215158 20339581 PMC2841246

[87] Iachininoto MG (2013). Cyclooxygenase-2 (COX-2) Inhibition Constrains Indoleamine 2,3-Dioxygenase 1 (IDO1) Activity in Acute Myeloid Leukaemia Cells. Molecules, 18, 10132–10145. 10.3390/molecules180910132 23973990 PMC6270179

[88] Barbieri S.S., Eligini S., Brambilla M., Tremoli E., Colli S. Reactive oxygen species mediate cyclooxygenase-2 induction during monocyte to macrophage differentiation: critical role of NADPH oxidase. Cardiovasc. Res. 2003;60:187–197. 14522422 10.1016/s0008-6363(03)00365-1

[89] Koki AT and Masferrer JL. Celecoxib: a specific COX-2 inhibitor with anticancer properties. Cancer Control. 2002;9(2):28–35. 11965228 10.1177/107327480200902S04

[90] Beg S, Swain S, Hasan H, Barkat MA, Hussain MS. Systematic review of herbals as potential anti-inflammatory agents: Recent advances, current clinical status and future perspectives. Pharmacognosy Reviews.2011;5(10):120–137. 10.4103/0973-7847.91102 22279370 PMC3263046

[91] Gebresamuel N, Gebre-Mariam T. Comparative Physico-Chemical Characterization of the Mucilages of Two Cactus Pears (Opuntia spp.) Obtained from Mekelle, Northern Ethiopia. Journal of Biomaterials and Nanobiotechnology. 2012; 3: 79–86.

[92] Harrabi B, Athmouni K, Hamdaoui L, Ben Mahmoud L, Hakim A, El Feki A, Zeghal K & Ghozzi H. Polysaccharides extraction from Opuntia stricta and their protective effect against HepG2 cell death and hypolipidaemic effects on hyperlipidaemia rats induced by high-fat diet, Archives of Physiology and Biochemistry. 2017; 123(4):225–237. 10.1080/13813455.2017.1307413 28372462

[93] Saravanakumar A, Ganesh M, Peng MM, Sh Aziz A & Tae Jang H. Comparative antioxidant and antimycobacterial activities of Opuntia ficusindica fruit extracts from summer and rainy seasons. Frontiers in Life Science.2015; 8:2, 182–191.

[94] Alves FAL, de Andrade AP, Bruno RdeLA, Silva MGdeV, de Souza MdeFV, Pessoa C, et al Genetic diversity and seasonal chemical profile by 1H NMR and cytotoxic activity in Opuntia and Nopalea genres. Journal of Medicinal Plants Research. 2016; Vol. 10(40), pp. 732–747, 25.

[95] Prabhakaran D, Senthamilselvi MM and Rajeshkanna A. Anticancer activity of Opuntia stricta (Flowers) against human liver cancer (HEPG2) cell line. Journal of Pharmacy Research. 2017; 11(7),793–797.

[96] Nowacki L., Vigneron P., Rotellini L., Cazzola H., Merlier F., Prost, et al. Betanin-Enriched Red Beetroot (Beta vulgaris L.). Extract Induces Apoptosis and Autophagic Cell Death in MCF-7 Cells. Phytotherapy Research. 2015; 29: 1964–1973. 10.1002/ptr.5491 26463240

[97] Gandia-Herrero F, Escribano J and Garcia-Carmona F. Biological activities of plants pigments Betalains. Critical Reviews in Food Science and Nutriton. 2016; 56: 937–945. 10.1080/10408398.2012.740103 25118005

[98] Sreekanth D, Arunasree MK, Roy KR, Chandramohan Reddy T, Reddy GV, Reddanna P. Betanin a betacyanin pigment purified from fruits of Opuntia ficus-indica induces apoptosis in human chronic myeloid leukemia Cell line-K562. Phytomedicine. 2007; 14(11): 739–746. 17482444 10.1016/j.phymed.2007.03.017

